# A systems approach to a spatio-temporal understanding of the drought stress response in maize

**DOI:** 10.1038/s41598-017-06929-y

**Published:** 2017-07-26

**Authors:** Zhenyan Miao, Zhaoxue Han, Ting Zhang, Siyuan Chen, Chuang Ma

**Affiliations:** 1 State Key Laboratory of Crop Stress Biology for Arid Areas, College of Life Sciences, Northwest A&F University, Yangling, 712100 Shaanxi China; 2 Center of Bioinformatics, College of Life Sciences, Northwest A&F University, Yangling, 712100 Shaanxi China; 3 Key Laboratory of Biology and Genetics Improvement of Maize in Arid Area of Northwest Region, Ministry of Agriculture, Northwest A&F University, Yangling, 712100 Shaanxi China

## Abstract

Crops are often subjected to periods of drought stress during their life cycle. However, how stress response mechanisms contribute to the crosstalk between stress signaling pathways and developmental signaling pathways is still unknown. We built a gene co-expression network from a spatio-temporal transcriptomic map of the drought stress response in maize (*Zea mays*), profiled from three tissues and four developmental stages and characterized hub genes associated with duplication events, selection, and regulatory networks. Co-expression analysis grouped drought-response genes into ten modules, covering 844 highly connected genes (hub genes). Of these, 15.4% hub genes had diverged by whole-genome duplication events and 2.5% might then have been selected during natural domestication and artificial improvement processes, successively. We identified key transcription factor hubs in a transcriptional regulatory network, which may function as a crosstalk mechanism between drought stress and developmental signalling pathways in maize. Understanding the evolutionary biases that have evolved to enhance drought adaptation lays the foundation for further dissection of crosstalk between stress signalling pathways and developmental signalling pathways in maize, towards molecular design of new cultivars with desirable yield and greater stress tolerance.

## Introduction

Drought or water deficiency is one of the most serious environmental stresses affecting plant growth and development and crop yield and productivity^1, 2^. To cope with drought stress, plants have evolved complex adaptive mechanisms, including regulation of transcription and gene expression, epigenetic plasticity, and metabolic reprogramming^3, 4^. Although tremendous progress has been made in understanding drought stress responses and tolerance mechanisms in the model plant *Arabidopsis thaliana* ^3, 5–7^, transfer of this knowledge from model species to crop plants is still inadequate^8, 9^. Compared with *Arabidopsis*, distinct stress-response mechanisms may be employed by crop species owing to different evolutionary processes, intense artificial selection, and more complex drought-responsive gene regulation networks and metabolic pathways. Therefore, extensive studies are required to systematically understand drought-stress-related mechanisms in crops, which will accelerate the development of new crop varieties with improved stress resistance aimed at achieving agricultural sustainability and food security for a growing world population.

Maize (*Zea mays*), serving as a raw material for the production of food, feed, and biofuel, is one of the world’s most important crops for humans and other animals. Drought stress is a substantial threat to maize production worldwide. Meanwhile, drought tolerance is a complex quantitative trait that is potentially correlated with other developmental traits, such as plant height, leaf area, stem diameter, and plant biomass^10^. These traits are generally quantitative, and each is controlled by multiple quantitative trait loci (QTLs) with relatively small individual effects on the corresponding traits, thus making them difficult to dissect by classical genetics approaches^11^. Because of their agricultural importance, drought-stress-related mechanisms in maize have been explored using high-throughput experimental techniques, including genome-wide association studies (GWAS)^12–17^ and next-generation sequencing (NGS)^18, 19^. The former identified DNA polymorphisms significantly associated with drought tolerance in maize. Nevertheless, the challenge in GWAS is the identification of causal variants for polygenic traits that are caused by variants related to multiple genes^20^. Meanwhile, NGS has led to the generation of omics data (transcriptomics, proteomics, and metabolomics), which has contributed to an understanding of the complex molecular regulatory networks associated with adaptation and tolerance to drought stress.

With the development of NGS, RNA sequencing (RNA-seq) has been widely used to interpret the dynamic reprogramming of the maize transcriptome during tissue and plant development under drought stress^18, 19, 21, 22^. Most of the previous studies were performed separately in different tissues and at different developmental stages. As a result, little is known regarding how drought adaptation in maize is controlled and regulated genetically and how stress signalling pathways crosstalk with developmental signalling pathways. Moreover, even though stress-response transcriptomic data in maize has been applied to detect drought-stress-induced alternative splicing (AS) events^23^, consideration of maize genome evolution, artificial selection, and known gene regulatory information remains largely absent. Although much progress has been made since the initial application of this technology^24^, there is still potential for it to be further applied in the systems biology field so as to advance our understanding of the mechanisms of the drought stress response in maize^28, 26^. In addition, with the rapid accumulation of data in public repositories, new challenges arise from the urgent need to effectively integrate many different omics datasets in studying the biological complexity of drought stress tolerance in maize^27^.

Using 94 Illumina RNA-seq data sets from maize leaf, ear, and tassel tissues under both well-watered and drought conditions^23^, we performed a system-wide investigation to address several key topics of fundamental importance for understanding drought adaptation in maize. Firstly, we constructed a gene co-expression network from drought-induced transcriptome profiles of three tissues across four developmental stages and identified modules of putatively co-regulated genes within this network. We asked whether these modular genes had specific expression patterns consistent with a role in developmental and tissue differentiation. Secondly, we identified hub genes within modules and subsequently examined the predicted functions of these genes in more detail to determine whether they contribute to crosstalk of signalling pathways between development and drought stress in maize. Finally, we studied drought-stress-related genes identified by previous population genetics research^28^, with the goal of learning more about evolutionary selection for drought adaptation, and to determine whether there is any evidence that these genes have biased evolution and, if so, whether that bias varies with the progression of selection.

## Results

### Generating a spatio-temporal transcriptional map of the drought stress response in maize

**Figure 1:**
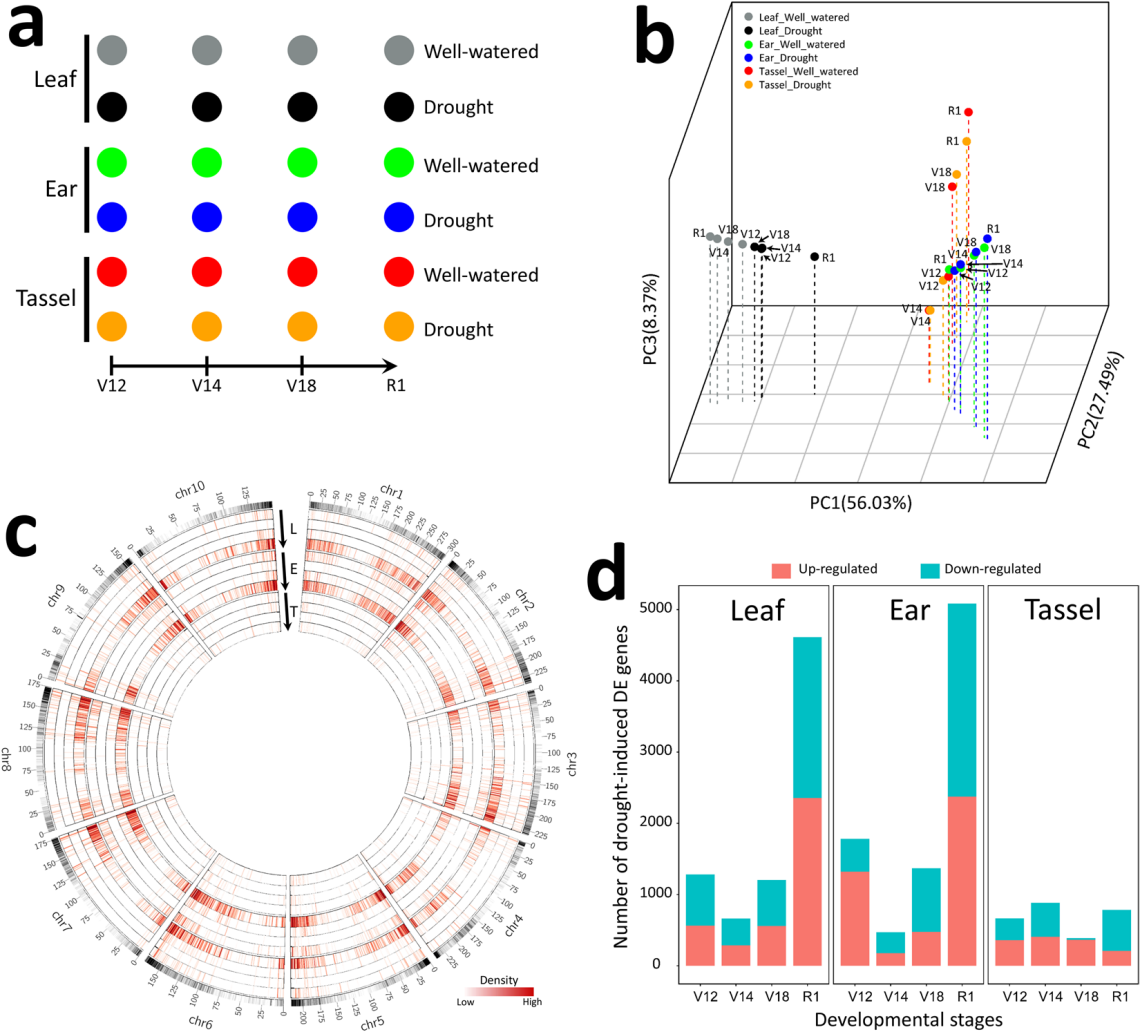
Drought treatment caused dynamic changes in maize growth: generation of a spatio-temporal gene expression map of the response. **(a)** Time-lapse procedure of maize sample treatment. **(b)** Principal component analysis of the different samples comprising the spatio-temporal map of drought response. **(c)** Density of differentially expressed (DE) genes of the drought stress response. Outer track shows density of total DE genes. Arrows indicate the four developmental stages from V12 to R1 in the three tissues, respectively. L, leaf; E, ear; T, tassel. **(d)** Number of genes that showed differential expression at four developmental stages and in three tissues after drought treatment.

A set of spatio-temporal transcriptomes from three B73 maize tissues (ear, tassel, and leaf) that spanned four developmental stages (V12, V14, V18, and R1) under control (well-watered) and drought stress conditions (Fig. 1a) were downloaded from NCBI’s GEO database (GSE71723). We mapped about 1,480 million quality-filtered reads (Table S1) to the maize B73 reference genome (RefGen_v3, ftp://ftp.ensemblgenomes.org/pub/release-29/plants/fasta/zea_mays/dna) using TopHat^29^. The resulting unique read alignments were further input into Cufflinks^30^ software to estimate gene expression abundance in terms of FPKM (fragments per kilobase per million). Using the commonly used criteria of defining an ‘expressed’ gene (FPKM ≥ 1), we found that 19,293–23,053 genes were expressed under the experimental conditions analysed (Fig. S1). A total of 27,885 genes were expressed under at least one experimental condition, 52.24% of which (14,625/27,885) were expressed under all 24 experimental conditions (Table S2). To investigate the contribution of different factors (tissue, developmental stage, and drought stress) to the global transcriptome dynamics, we performed principal component analysis (PCA) on FPKM values of the 27,885 genes expressed in at least one experimental sample. The three-dimensional PCA plot showed that tissue type contributed more to the global differences in gene expression than did the other two factors (developmental stage and drought stress). As shown in Fig. 1b, leaf samples were clustered far away from ear and tassel samples, indicating that the expression profiles of leaf tissues were markedly different from those of ear and tassel tissues. Similarly, ear expression profiles were also very different from those of tassel tissues at the V14, V18, and R1 stages (Fig. 1b). Interestingly, we observed that in leaf tissue, transcriptome fluctuations induced by drought stress were more dramatic than those induced by developmental stage; in the other two tissues (ear and tassel), this pattern was not apparent. Nevertheless, we observed that transcriptomic profiles in ear and leaf tissues were markedly affected by drought stress at some developmental stage (e.g. R1 stage). These spatio-temporal dynamics of the transcriptome in the maize response to drought stress were also revealed by hierarchical clustering analysis based on FPKM values of the 27,885 expressed genes (Fig. S2).

We further performed statistical tests between gene expression values profiled from each drought-stressed sample and the corresponding control (e.g. leaf&V12&well-watered versus leaf&V12&drought) using Cuffdiff^30^. As a result, we identified 11,284 differentially expressed (DE) genes (false discovery rate [FDR]-adjusted *P*-value ≤ 0.05 and fold change ≥2 or ≤0.5), of which 87.1% (9,830) were also identified as DE genes using DEseq^31^ and/or edgeR^32^ (Table S3; Fig. S3). The density of DE genes identified in each statistical test was greater on the distal portion of the chromosomes compared with the regions around the centromeres (Fig. 1c). Differences in the density of DE genes were evident in different tissues at the four developmental stages from V12 to R1 (Fig. 1c), which also suggests a spatio-temporally dynamic pattern of the maize response to drought stress. Tassel tissue had the smallest number of DE genes (665 for V12, 885 for V14, 387 for V18, and 784 for R1), which indicated a limited response under drought stress at the four developmental stages analysed (Figs S4 and S5). By contrast, ear and leaf tissues had relatively large numbers of DE genes, corresponding to 5,084 and 4,614 genes at the R1 stage, respectively (Figs 1d, S4, S6, and S7). Gene ontology (GO) analysis of DE genes identified in each statistical test revealed enrichment for specific biological processes, in specific tissues and at specific developmental stages (Table S4). For instance, the GO term “photosynthesis” was specifically enriched in ear tissue at the V12 stage and in tassel tissue at the R1 stage; the GO term “metal ion transport” was only enriched in tassel tissue at the V14 stage. Taken together, these preliminary results revealed the spatio-temporal characteristics of transcriptome regulation in the maize response to drought stress.

### Drought-stress-related gene modules within the spatio-temporal transcriptional map

**Figure 2:**
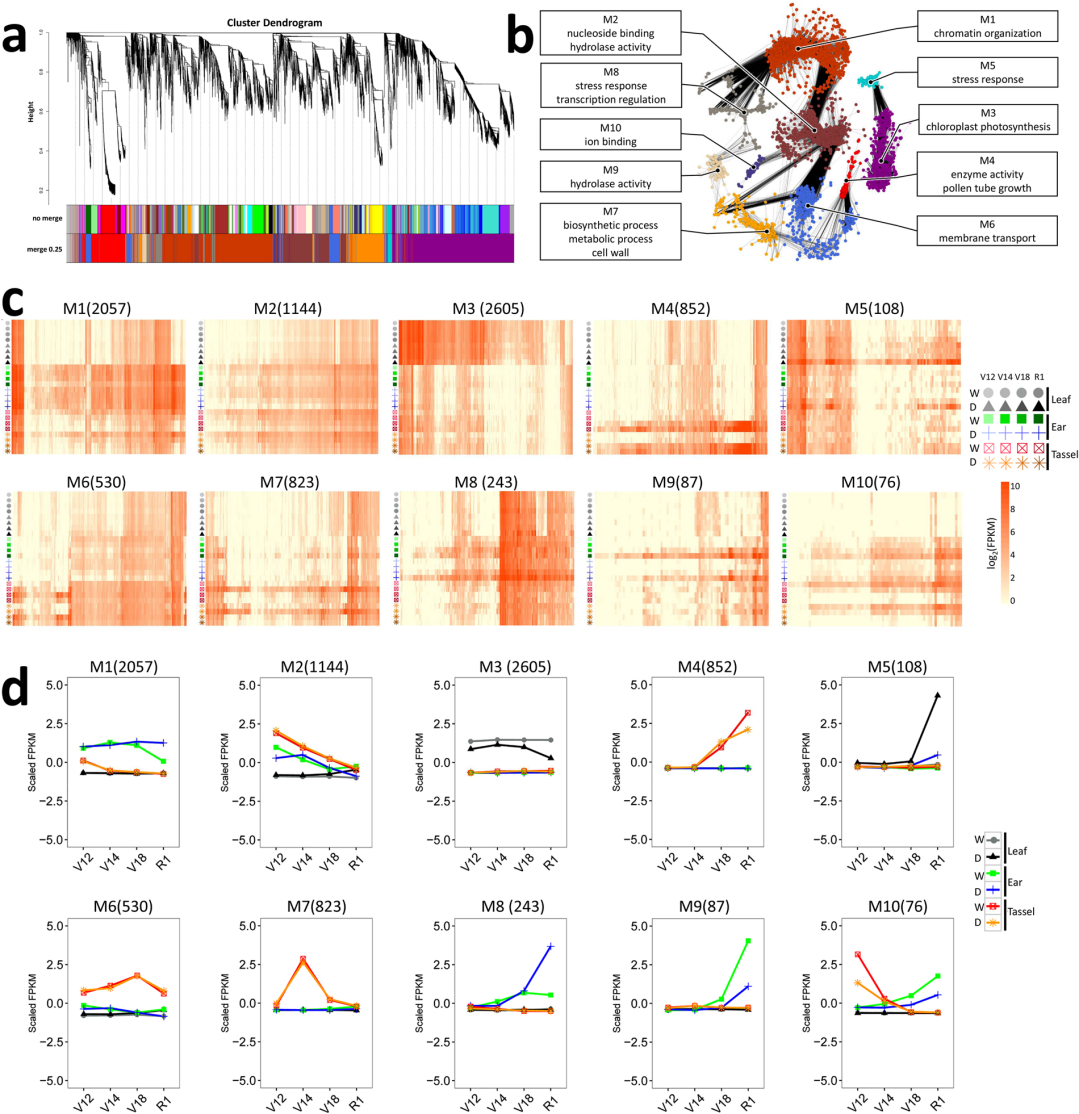
Co-expression network analysis identifying gene modules underlying drought stress at four developmental stages in three tissues. **(a)** Hierarchical cluster dendrogram showing co-expression modules identified using weighted gene co-expression network analysis (WGCNA) of the differentially expressed genes. Modules corresponding to branches are labelled with colours indicated by the colour bands underneath the tree. With 0.25 threshold merging, 10 modules were generated. **(b)** Connection network among the 10 modules. Nodes are colour-coded by module. The over-represented gene ontology (GO) terms for each module are shown. **(c)** Heatmap showing gene expression levels of the genes within the 10 modules in three tissues across four developmental stages. W, well-watered; D, drought. **(d)** Changes in expression of the genes within the 10 modules over the course of development in three tissues. W, well-watered; D, drought.

To reveal crucial shifts in gene networks in maize under drought stress, we further used a systems biology approach, namely weighted gene co-expression network analysis (WGCNA), to perform a network-level analysis of co-expression relationships among 11,284 DE genes across 24 experimental conditions. WGCNA identifies clusters (modules) of highly co-expressed genes based on gene expression similarity, and has been demonstrated to identify important genes associated with complex phenotypes and biological processes in plants^33–35^. We identified 10 gene modules (designated M1–M10; capturing 8,525 genes) (Fig. 2a,b) corresponding to clusters of 76 to 2,605 highly co-expressed genes (Fig. 2c; Table S3).

Module M1 formed a large cluster of 2,057 DE genes enriched for functions related to chromatin organization (Fig. 2b; Table S5). Hierarchical clustering of genes in module M1 showed separation of ear from the other two tissues (leaf and tassel) throughout all developmental stages (Fig. 2d), owing to higher expression in ear tissue (mean value of scaled FPKM >0) than in leaf and tassel tissues (mean value of scaled FPKM <0) across the four developmental stages analysed. This result indicated that in module M1, ear-specific gene expression markedly dominates over stage-specific gene expression as well as drought-stress-specific gene expression. The dominance of tissue-specific gene expression was also observed in modules M3 (leaf-specific) and M6 (tassel-specific), which were enriched for functions associated with chloroplast photosynthesis and membrane transport, respectively (Fig. 2b; Table S5).

Module M4 included 852 genes linked to enzyme activity and pollen tube growth (Fig. 2b; Table S5). Genes in this module underwent dramatic up-regulation immediately after the V14 stage in tassel tissue and remained up-regulated at later stages. By contrast, in module M7, expression levels of genes involved in biosynthetic processes, metabolic processes and cell wall increased quickly after the V12 stage but then decreased quickly after the V14 stage and remained low at later stages (V18 and R1). Specific expression patterns and distinct functional enrichments were also observed in modules M8 and M9, in which genes were involved in stress response, transcription regulation and hydrolase activity (Fig. 2b; Table S5) and exhibited increased expression patterns in ear tissue after the V14 stage. Stress- and regulation-related genes were up-regulated by drought stress, whereas hydrolase-activity-related genes were down-regulated by drought stress.

Likewise, in module M5, related to stress response in leaf tissue (Table S5), genes underwent dramatic up-regulation during drought stress after the V18 stage. Interestingly, expression of genes in module M2, related to nucleoside binding and hydrolase activity (Fig. 2b; Table S5), was significantly higher in the early V12 stage and then gradually decreased during the later three stages (V14, V18, and R1) in ear and tassel tissues. This suggests that drought-stress response genes in the M2 module might also be involved in differentiation between reproductive and vegetative tissues in early development. In the unique module M10, associated with ion binding (Fig. 2b; Table S5), genes exhibited opposite expression trends between ear and tassel tissues. This indicated that drought-stress-response genes in module M10 might also play critical regulatory roles that lead to further tissue development and differentiation.

Moreover, we investigated how different AS types were distributed among these 10 modules. In total, 6,068 AS events were identified, of which intron retention (IR) was the most abundant type (42.5%), followed by alternative acceptor (AA; 15.4%), alternative donor (AD; 10.6%), exon skipping (ES; 6.97%), and other types (Table S6). These results were similar to the frequency of AS observed in *Arabidopsis* and soybean^36, 37^. The variations in the frequency of the different events among the 10 modules were very slight, except that the proportions of ES events in modules M5, M8, M9, and M10 were relatively lower than those in the other modules. This phenomenon might be related to the differentiation of module functions, such as stress response, transcription regulation, hydrolase activity, and ion binding.

### Identification of hub genes within network modules

In each network module, we observed that a portion of the genes had extremely high connectivity with other genes. These highly connected genes (usually referred to as hub genes) were thought to be critical components of the network, because of their central location within the corresponding modules. Therefore, we set out to identify hub genes using the network analysis package igraph^37^. As a result, the 10% (844) of genes with the highest hub scores were identified as hub genes (Table S7; Fig. S8). Regression analysis between expression levels and developmental stages indicated that the majority of hub genes (88.51%; 747/844) may be involved in the coordination of developmental and drought stress responses in maize (Table S8).

Among 844 hub genes, there were 49 transcription factors (TFs) represented, from distinct families, some of which (*bHLH*, *bZIP*, *C2H2*, *Dof*, *MYB*, *ERF* and *NAC*) have been reported to have roles in plant development and drought stress responses in *Arabidopsis*, maize, and other plants^39–44^. Regression analysis suggested that the expression of 44 TFs was significantly associated with the four developmental stages, which also indicated their potential roles in both the developmental and drought stress responses in maize (Table S8). We also noted that some TFs from G2-like and GATA families may be coordinated with chloroplast development, photosynthesis, and the drought stress response^45, 46^.

**Figure 3:**
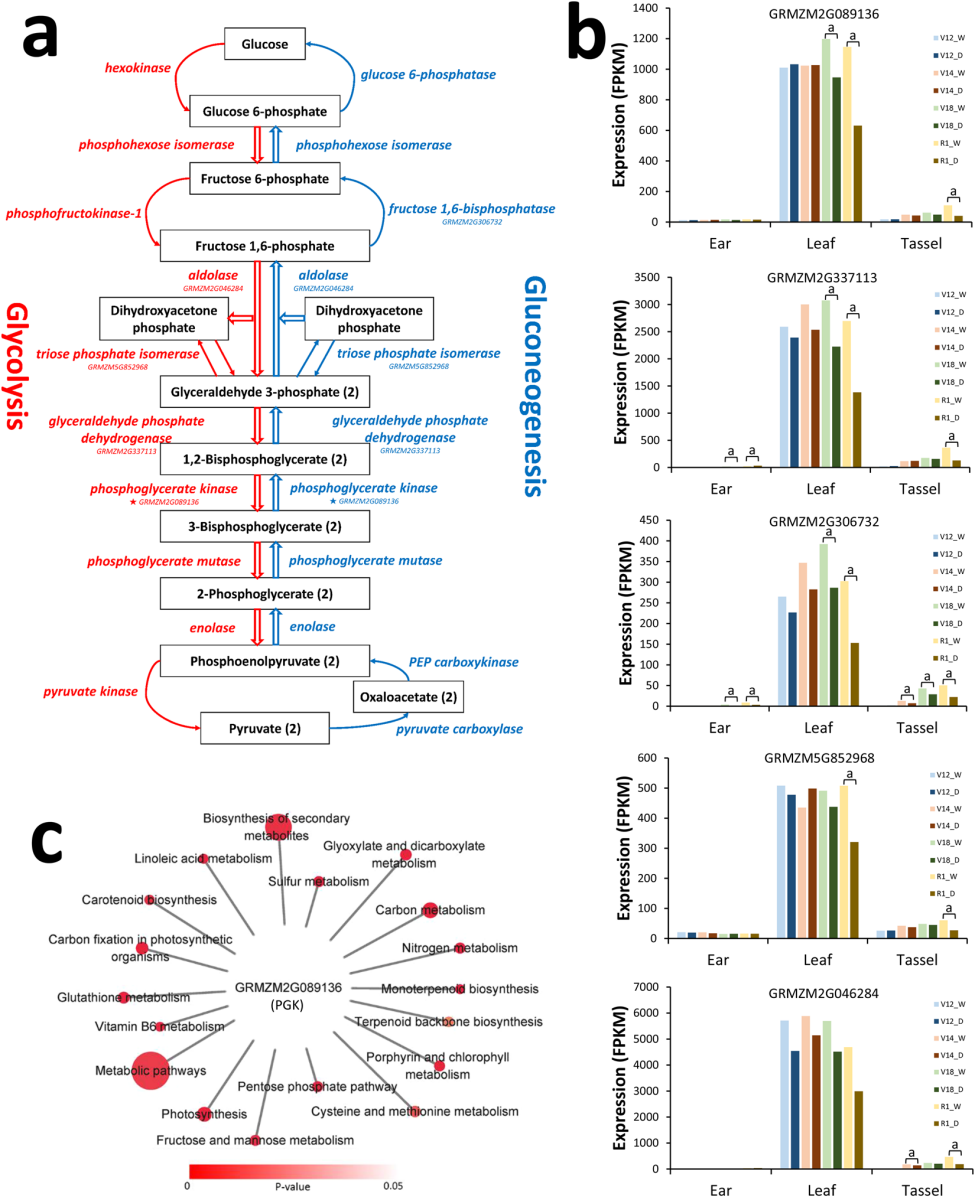
The dynamic response of hub genes related to the glycolysis pathway. **(a)** Map of the glycolysis pathway (red) and reversible gluconeogenesis pathway (blue). Network gene symbols are shown beneath gene names; asterisks indicate a hub gene. **(b)** Expression profiles of network genes associated with the glycolysis pathway. W, well-watered; D, drought. a, Adjusted *P*-value ≤ 0.05. **(c)** Pathway enrichment of phosphoglycerate kinase (PGK) neighbourhoods.

Besides TFs, there were 703 other hub genes that may also have roles in both the developmental and drought stress responses (Table S8). Of note, six hub genes (*GRMZM2G079381*, *GRMZM2G089136*, *GRMZM2G099984*, *GRMZM2G436199*, *GRMZM2G154437*, and *GRMZM2G164325*) have recently been reported to show DNA polymorphisms, and to be candidate genes associated with maize grain yield and display phenotypic traits related to drought stress^13–17, 47^. Another interesting hub gene, *GRMZM2G089136*, encodes a phosphoglycerate kinase (PGK) protein involved in the Calvin–Benson–Bassham cycle as well as in the glycolysis and gluconeogenesis pathways (Fig. 3a). The expression of *GRMZM2G089136* was highest in leaf tissue and was only down-regulated in drought-stressed leaf samples at the V18 and R1 stages (Fig. 3b). This indicated that the glycolysis and gluconeogenesis pathways might play a crucial role during leaf development. Meanwhile, the low abundance of PGK expression may be connected to a decrease in carbon fixation caused by the reduction of photosynthesis during drought stress, indicated by the functional enrichment of PGK-connected genes, including those involved in nitrogen metabolism, sulphur metabolism, linoleic acid metabolism, monoterpenoid biosynthesis, and glutathione metabolism (Fig. 3c; Table S9).

### Expression and functional analysis of hub genes associated with duplication events

**Figure 4:**
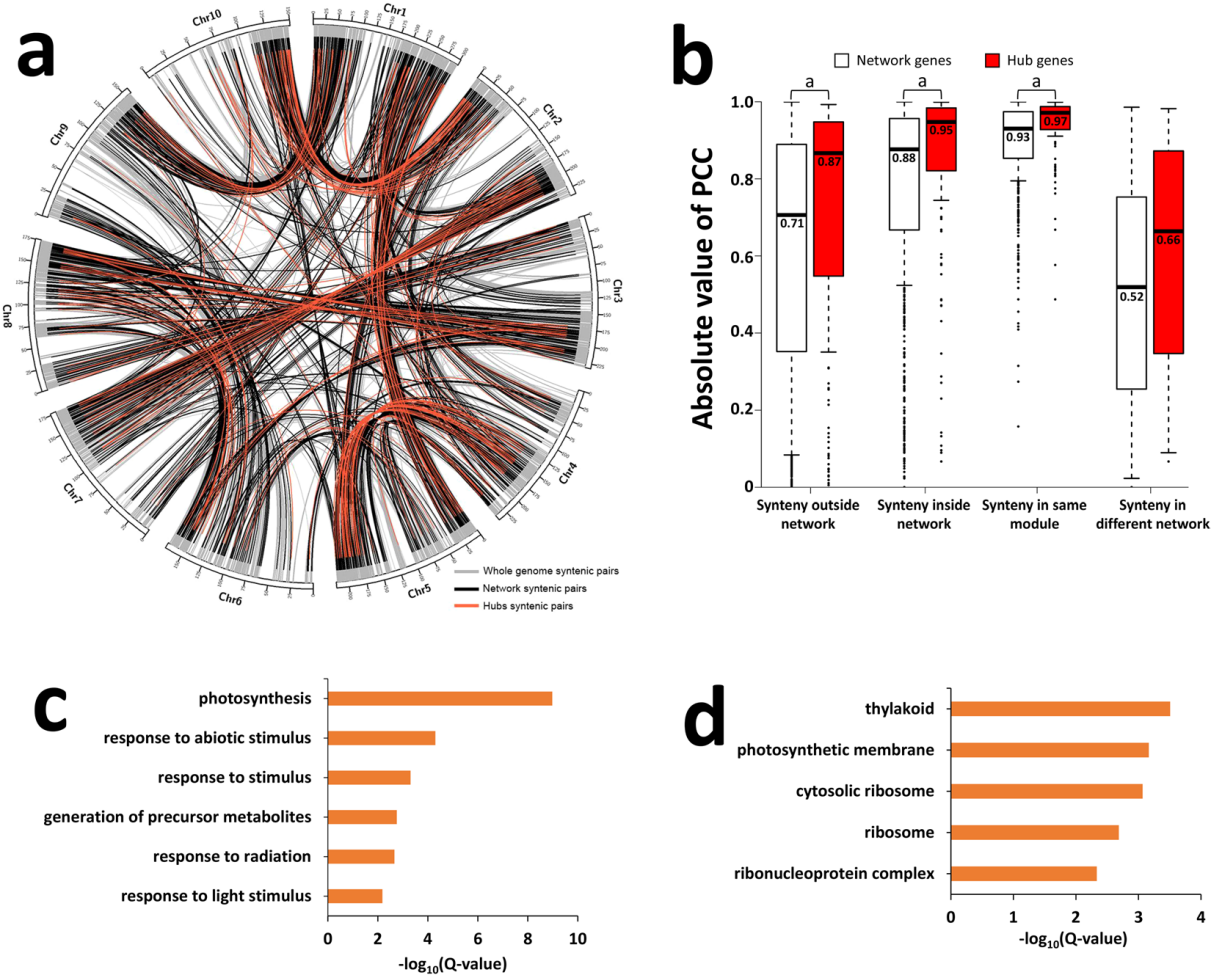
Syntenic analysis of hub genes revealed evolutionary biases for drought adaptation in maize. **(a)** Distribution of genome-wide syntenic gene pairs. Grey indicates whole-genome syntenic pairs. Black indicates syntenic pairs in the same network. Red indicates syntenic hub gene pairs. **(b)** Comparison of Pearson’s correlation coefficient (PCC) values between syntenic genes in network sets and hub sets. The statistical analysis was conducted between each set of network pairs and hub pairs by Student’s t-test. a, P < 0.01. **(c)** Gene ontology enrichment for conserved hub genes. **(d)** Gene ontology enrichment for diverged hub genes.

Duplication events, such as large-scale (segmental or whole) genome duplications, are common phenomena in plant genome evolution, resulting in the expansion and diversification of many gene families^48^. Post-duplication, many duplicated genes are retained in the genome as homologous gene pairs (HGPs), in which the individual genes may be subfunctionalised (partitioning and sharing the original gene function) and/or neofunctionalised (gaining novel functions) via sequence and/or expression divergence^49, 50^. Duplication events have resulted in the generation of 1,242 syntenic blocks in the whole maize genome identified using the SynMap^51^ application within CoGe (Fig. 4a; Table S10). This raised the question of whether drought-responsive hub genes were associated with duplication events.

We found that 35.07% (296/844) of hub genes were distributed in 424 syntenic blocks, forming 312 HGPs (Table S11). These 312 HGPs could be classified into three groups: 112 HGPs with two paired duplicated genes within the same module (SM group), 37 HGPs with two paired duplicated genes from different modules (DM group) and 138 HGPs with one duplicated gene outside of the network (ON group). The similarity in gene expression of duplicated hub genes was calculated using the Pearson’s correlation coefficient (PCC) algorithm. The mean PCC value of HGPs in the SM group was 0.97. In contrast, for HGPs in the DM and ON groups, much lower PCC values of 0.66 and 0.87, respectively, were obtained (Fig. 4b). Similar patterns of expression divergence for HGPs in the SM, DM and ON groups were also observed for non-hub network genes (Fig. 4b). However, non-hub network genes tended to have greater expression divergence than hub genes. This is in accordance with the expectation that duplicated hub gene pairs in the SM group have more highly conserved expression patterns. In addition, we observed divergence in developmentally regulated trends between these hub HGPs in leaf, ear and tassel tissues under both well-watered and drought conditions (Table S11). These differences can be clearly seen in some TFs, such as *GRMZM2G020054* (ethylene response factor; ERF), *GRMZM2G101350* (basic helix-loop-helix; bHLH) and *GRMZM2G003406* (MYB) (Fig. S9). These changes in HGPs might be a result of neofunctionalization induced by whole-genome duplication and could affect drought tolerance as development progresses to later stages.

To examine whether HGPs with more highly conserved expression patterns shared more similarity of function, we performed GO enrichment analysis on hub genes belonging to the SM, DM and ON groups, respectively. Conserved hubs (SM group) were enriched in several biological processes, including photosynthesis and response to abiotic stress (Fig. 4c). By contrast, diverged hubs (DM and ON groups) were mainly enriched in cellular components, such as thylakoid, photosynthetic membrane and ribosome (Fig. 4d). Overall, we observed that hub genes with duplicated copies in the same modules exhibited less divergence in expression and more functional conservation during the evolutionary process, which may be required to maintain their dosage balance and strong connections with other genes for coordinating developmental and stress responses in maize.

### Expression and functional analysis of hub genes related to maize domestication and improvement

Maize was domesticated approximately 10,000 years ago in southwestern Mexico and subsequently, has been subjected to intensive improvement efforts, culminating in hybrid lines that are highly adapted to modern agricultural practices^52, 53^. Domestication and improvement involved a radical phenotypic transformation from the wild progenitor, resulting in various adaptations for tolerance of abiotic stresses. In a recent comparative population genomics study, 468 and 574 candidate genes were identified as likely targets of selection during maize domestication and improvement, respectively^28^. We therefore asked whether there were any drought-responsive hub genes associated with maize domestication and improvement.

**Figure 5:**
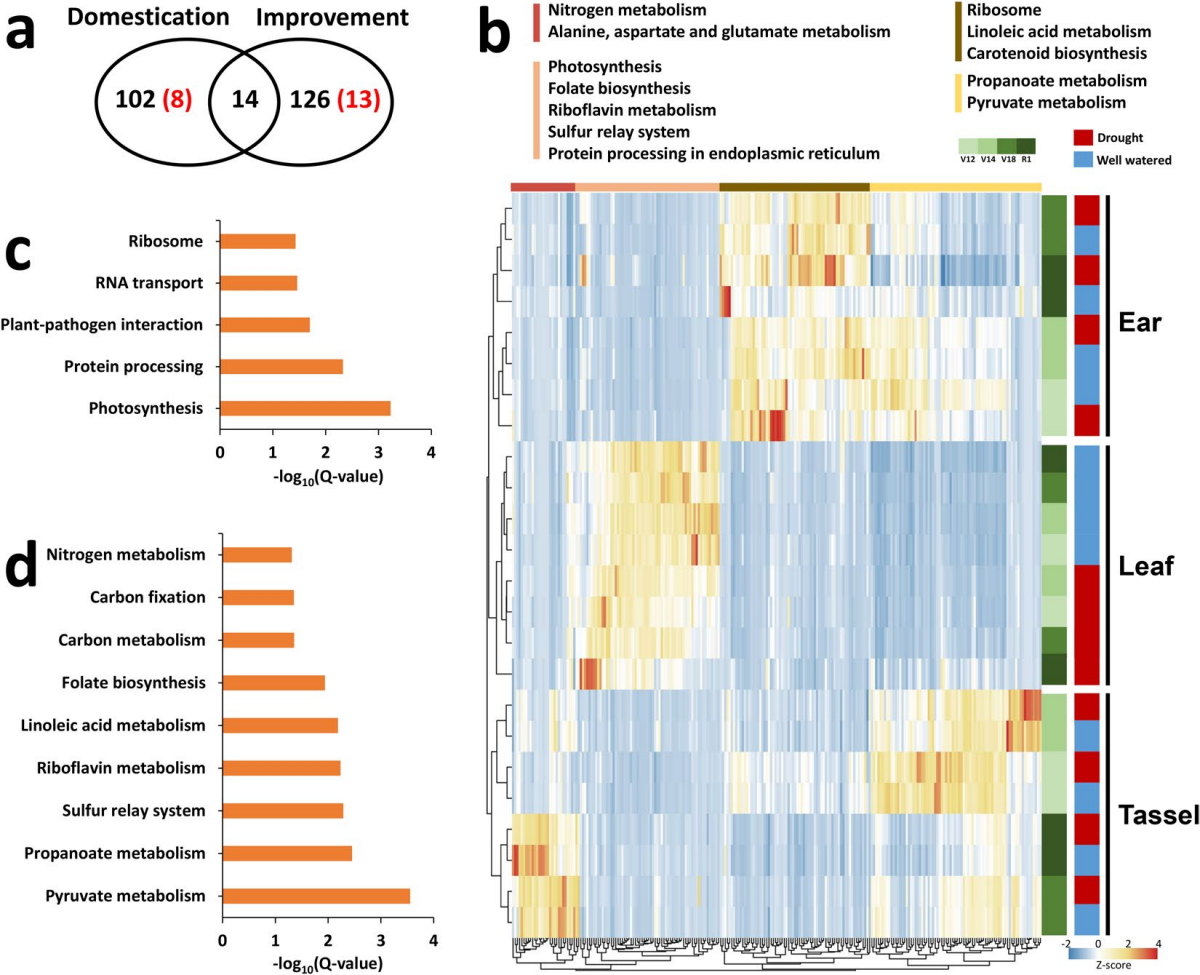
Evidence for maize-specific domestication and improvement. **(a)** Numbers of domestication genes and improvement genes and their overlap. Red numbers in parentheses indicate hub genes. **(b)** Heatmap comparing scaled expression values of domestication and improvement genes at four developmental stages in three tissues. Top colour bars indicate pathway enrichment (Q < 0.05) for tissue-specific gene sets. **(c)** Gene ontology enrichment for domestication genes. **(d)** Gene ontology enrichment for improvement genes.

We observed that 116 (8 hub) and 140 (13 hub) network genes were associated with maize domestication and improvement, respectively (Fig. 5a; Table S12). Hierarchical clustering of expression patterns of these selective genes revealed tissue specificity (Fig. 5b). Interestingly, the dominance of drought-specific gene expression over stage-specific gene expression was only observed in leaf tissue, but not in ear or tassel tissues, which partly suggested that spatial variation of drought stress response may have arisen from different selection events. To examine this hypothesis, we compared the over-representation of pathway categories between the 116 domestication genes and 140 improvement genes. We found that the domestication genes were enriched in five pathways, including photosynthesis, protein processing and plant–pathogen interaction (Fig. 5c). By contrast, the improvement genes were significantly enriched in several secondary metabolism pathways, such as pyruvate metabolism, propanoate metabolism and the sulphur relay system pathway (Fig. 5d). These findings illustrated that genes identified as likely targets of selection did have biased pathway association, which could be related to various phenotypic changes during maize domestication and improvement, respectively. Furthermore, we obtained further insight into the impact of hub genes, which might play major synergistic roles during domestication selection, from the presence in our network of two domesticated hub genes (*GRMZM2G102664* and *GRMZM2G138230*) related to pathways of transcription initiation, RNA transport and mRNA surveillance (Fig. S10). These hub genes are implicated in transcription functions that could be crucial for stress adaptation in domestication processes. This observation provided strong evidence for the previous notion that major phenotypic changes in crop plants during domestication are driven by changes in transcription functions^54, 55^. Moreover, among these 21 hub genes identified as likely targets of selection, 12 genes were duplicated and 19 genes were significantly associated with developmental stages, which also indicated their potential roles in both developmental and drought stress responses during maize evolution (Tables S13–S14).

### Analysis of the transcriptional regulatory network in maize during drought stress

**Figure 6:**
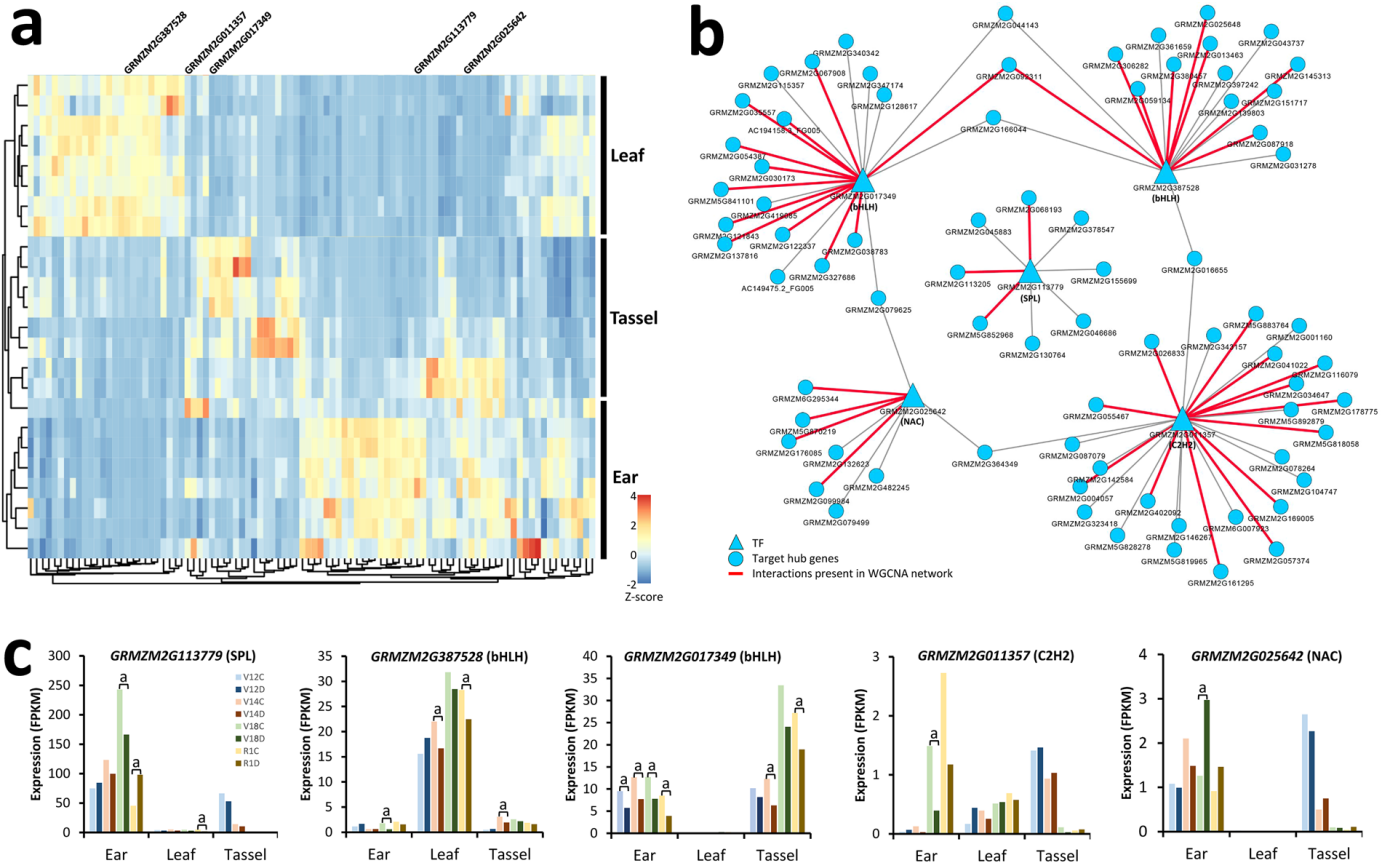
Dynamic changes in the transcriptional regulatory network in response to drought stress. **(a)** Heatmap comparing scaled expression values of 94 differentially expressed transcription factors (TFs) at four developmental stages in three tissues. The genes listed on the right (*GRMZM2G025642*, *GRMZM2G011357*, *GRMZM2G387528*, *GRMZM2G113779* and *GRMZM2G017349*) encode five network TFs with over-represented targets. **(b)** Topology of five TFs possessing over-represented targets in the network. TFs are represented as triangles and targets are represented as ellipses. Red colour indicates targets included in the network. Blue colour indicates targets outside of the network. **(c)** Expression profiles of *GRMZM2G025642*, *GRMZM2G011357*, *GRMZM2G387528*, *GRMZM2G113779* and *GRMZM2G017349* at four developmental stages in three tissues. a, Adjusted *P*-value ≤ 0.05.

To further explore the regulatory role of the 884 hub genes during maize development and stress responses, we constructed a transcriptional regulatory sub-network covering 6,796 regulatory interactions consisting of 229 TFs and 807 target hub genes (Fig. S11; Table S15). These regulatory interactions were inferred by combining TF binding motifs and regulatory elements, and were compiled from the literature by the authors of the PlantRegMap database^56^. We observed that 94 TFs were differentially regulated by drought treatment during development, 68 of which were covered by our co-expression network. Hierarchical clustering of expression patterns of these 94 drought-responsive TFs revealed tissue specificity (Fig. 6a), suggesting that these TFs might function in mediating signalling crosstalk between drought response and tissue development. In addition, we obtained 16 TF genes possessing a significantly over-represented target number (hypergeometric test, adjusted *P*-value ≤ 0.05; Table S16), among which, five key candidate TF genes (*GRMZM2G025642*, *GRMZM2G011357*, *GRMZM2G387528*, *GRMZM2G113779* and *GRMZM2G017349*) were involved in important regulatory functions in our co-expression network (Fig. 6b). Expression profiles of these five TFs demonstrated significant tissue-specific and stage-specific characteristics (Fig. 6c). For example, *GRMZM2G113779* (encoding SQUAMOSA promoter binding protein; SPL), known to be a major plant-specific TF related to flower development, was highly expressed in ear tissue and regulated by drought stress. The SPL gene family has recently been reported to function as a molecular link between small RNA signals, which might be required for the coordination of adult development and floral transition in *Arabidopsis* ^57^. This suggests the versatile roles of SPL gene family members in the crosstalk between various developmental pathways. Our work established a link between drought stress signalling and developmental signalling in maize at the tissue level.

Two genes encoding basic/helix-loop-helix (bHLH) TFs, namely *GRMZM2G387528* and *GRMZM2G017349*, were regulated in leaf and reproductive tissues, respectively. Several previous studies have indicated that bHLH proteins are important regulatory components in transcriptional networks in plant systems, controlling a diversity of processes from cell proliferation to cell lineage establishment^39–41^. We observed obvious opposite expression patterns in our spatio-temporal transcriptional data, indicating that these two TFs might be partly functionally differentiated. Two other TFs, corresponding to *GRMZM2G025642* (NAC) and *GRMZM2G011357* (C2H2), are frequently reported to play important roles in the responses of plants to abiotic stress and in plant development^42–44^. The expression of these two TFs was relatively stable in our samples. Nevertheless, several trends affected by drought stress and tissue specificity could still be observed. Furthermore, functional associations of these TFs both in stress-related processes and tissue development are discussed later.

## Discussion

In this work, we constructed a drought-responsive gene co-expression network from transcriptome profiles of three maize tissues across different stages of development and identified modules of putatively co-regulated genes within this network that offered the possibility for studying the spatio-temporal and environmental regulation of developmental processes in maize. Modules in our network showed functional specificity in tissues and were regulated dynamically by drought stress. Our investigation also identified central genes with strong connectivity in the network. Such hubs include genes with known or suspected roles in stress response and development. We showed that a proportion of hub genes might have diverged from whole-genome duplication events and then were then artificially selected during domestication and improvement processes, which drove the generation of major drought-tolerance traits by changes in transcriptional regulators. These observations not only provide new paradigms for understanding the evolutionary innovation driven by genome duplication and artificial selection, but also lay the foundations for further dissection of crosstalk between stress signalling pathways and growth and developmental signalling pathways in maize, leading in turn towards the molecular design of new cultivars with desirable yield characteristics and strong stress tolerance.

### Divergence in gene expression throughout maize developmental phases

Drought resistance in maize is highly complex and requires a vast interaction of genes and cooperation of processes. Confirming the phases of different processes has an important significance in explaining maize development and guiding the selection of tissue development stages for different purposes. Many studies related to maize development and drought resistance have been performed from the perspective of morphology, molecular biology and omics^58–60^, but the deep profiling in our study fills in many missing details.

Although ear and tassel tissues were well distinguished, common variance in expression was observed at the early V12 stage of development by PCA analysis (Fig. 1b), and expression of genes present in both ear and tassel at the V12 stage tended to be positively correlated (Fig. S2), indicating that the expression of large sets of genes changes coordinately in the early development of ear and tassel. It is reasonable to assume that some regulators are shared between the ear and tassel, so that the two relatively independent reproductive tissues can undergo the early development process at the same time. Previously, a global comparison of AS also suggested that substantial coordination of biological processes occurs in ear and tassel tissues^23^. However, regulators responsible for coordinating the early development of the ear and tassel remain to be determined. Several interesting clues were observed within our modular network. Modules are composed of genes that have similar patterns of expression across all samples. Our results indicated that tissue identity is a primary factor that explains transcriptome variation throughout maize development and suggest that developmental stage and drought stimuli have relatively minor effects on transcriptional profiles relative to tissue type. By examining the factors that explain transcriptome variation across development in ear and tassel tissues, we identified module M1, related to chromatin organization, preferentially expressed in ear tissue; we also identified modules M4, related to enzyme activity and pollen tube growth, M6, related to transport, and M7, related to biosynthetic and cell wall functions, preferentially expressed in tassel tissue. It is likely that the dynamic expression patterns of these functional genes across development stages contributed to the coordination and divergence between these two reproductive tissues. In addition, drought was found to cause massive changes during developmental processes in ear and leaf tissues but only minor changes in tassel tissue. We observed that drought-induced gene expression changes occurred in a developmental-stage-dependent manner. Thus, genes within modules M1, M2, M3, M4, M5, M8, M9 and M10 underwent dramatic regulation by drought stress during the V12 and R1 stages in comparison with the V14 and V16 stages. Meanwhile, drought response within these modules tended to be highly tissue-specific. In modules M1, M2, M8 and M9, stage-specific changes in drought response were observed in ear tissue, whereas in modules M3 and M5, stage-specific changes in drought response were observed in leaf tissue. Stage-specific changes in drought response of the tassel only occurred in module M4. These observations highlight the tissue-specific and stage-specific patterns of the drought-induced transcriptome profile. Nevertheless, the biological importance of key genes within our network will require further investigation. Taken together, our study provides information on when and where all of these genes are actively expressed. The spatio-temporal gene expression during drought stress, combined with predictions of gene function based on their annotation, is very useful for accelerating understanding of their roles.

### Hotspots for crosstalk between drought stress and developmental signalling pathways

Many genes with various functions have been identified as being involved in the response to drought stress in maize and other crops, but little is known about the relationships between these genes. In addition, more attention should be directed towards responses to the crosstalk between abiotic stress and developmental signalling because, in nature, crops co-exist with various abiotic stresses including drought. Thus, the current challenge for understanding the drought stress response is to discover the hub genes at a system-based level that coordinate the formation of this complex process. A large proportion of hub genes identified in our study have unknown functions. Nevertheless, co-expression network modules provide excellent inferences for uncharacterized genes through “guilt by association”. For example, *GRMZM2G089136*, belonging to module M4, is the maize orthologue of genes for PGK in *Arabidopsis* and rice, which may indicate that its functions in glycolytic and photosynthetic processes are similar to those described in rice and *Arabidopsis*, respectively^61, 62^. Glycolysis generates high-energy ATP compounds by anaerobic catabolism of glucose and acts in a fundamental role, providing other metabolic pathways with intermediate compounds and energy^63^. Sugars, aside from playing an important role as carbon and energy sources, also act as osmolytes that improve tolerance to drought conditions. By decreasing osmotic stress, they mitigate the reduction of cell turgor, also stabilizing membranes and subcellular structures^64^. Moreover, genes (*GRMZM2G046284*, *GRMZM2G306732*, *GRMZM2G852968* and *GRMZM2G337113*) for several other enzymes involved in this pathway were also observed in our network (Fig. 3a). In addition, the hub status of *GRMZM2G089136* suggested that the drought stress response seems to be the result of the activation of the photosynthetic pathway together with the glycolytic pathway, as well as of subsequent secondary metabolism such as sulphur metabolism, linoleic acid metabolism and glutathione metabolism.

On the other hand, as gene regulators, TFs are involved in myriad biological processes, such as growth, development, cell cycle progression and responses to environmental stimuli^65^. However, there have been few reports on TFs that function in the crosstalk between abiotic stress and developmental signalling pathways. We obtained two maize orthologs (*GRMZM2G387528* and *GRMZM2G017349*) of bHLH genes in *Arabidopsis* that exhibited different expression patterns among the three tissues, which indicates that they may function specifically in vegetative and reproductive tissues, respectively. In addition, a SPL ortholog (*GRMZM2G113779*), known to function in flower development, is likely to be involved in drought stress signalling pathways. It is an interesting phenomenon that genes for TFs were, not unusually, central to modules relative to genes with other functions. Among 844 hub genes, only 49 (5.8%) genes were identified as genes for TFs and, in contrast, 807 (95.6%) genes may be regulated by these TFs. We propose two explanations for this observation. Firstly, precise regulation of module genes may arise through the combinatorial protein interactions of TFs. Secondly, TFs may act transiently to establish the regulative relationships early in development, and our study did not capture this time point. For example, one NAC orthologue (*GRMZM2G025642*) and one C2H2 orthologue (*GRMZM2G011357*) are well known to function in stress response and plant development. However, the expression level of these two TFs was low among our samples, and the connectivity was relatively poor as well (Fig. S12). Nevertheless, the interaction among these TFs could offer a multi-coupler for crosstalk between drought stress and developmental signalling pathways.

### Evolutionary biases for drought adaptation in maize

Maize has experienced several rounds of duplication events during its genome evolution. Post-duplication, the accumulation of many duplicated genes with subfunctionalisation or novel functions for stress adaptation has occurred. We observed that hub genes involved in drought response are preferentially retained as duplicates with conserved functions. This phenomenon could be explained by the gene balance hypothesis that any successful genome has evolved, by many stepwise selections, an optimum balance of gene products binding with one another to produce multi-subunit complexes. If a connected gene pair is fractionated, dosage imbalance is expected and disease results. Connected genes should tend to be retained as pairs only because they are costly to remove^48^. Thus, genes acting as hubs in our gene-interaction networks are more likely to be retained without change of function during ever-present purifying selection. In addition, in our study, genes selected by domestication and improvement were enriched in distinct pathways, respectively. It is known that, in response to any biotic or abiotic stress, maize may evolve adaptations that provide resistance or tolerance. Theory developed by a previous study has suggested that resistance traits reduce the level of damage by the stressor, whereas tolerance traits reduce the negative fitness impact for a given amount of stress^66^. The logic behind this theory is that natural selection for resistance results in low levels of stress, and hence reduced selection for tolerance. Conversely, organisms with a high level of tolerance should not experience selection for resistance, because stress does not reduce fitness^66^. Thus, we deduced that pathways involved in drought resistance such as photosynthesis, protein processing and plant–pathogen interaction might be naturally selected for by domestication events. Subsequently, genetic improvement technologies have artificially enhanced some secondary metabolic pathways related to drought tolerance in maize.

The evolutionary biases of drought adaptation in maize were not only reflected by different pathways selected by domestication and improvement, respectively, but also indicated by the distinct expression patterns of genes within individual tissues. It appears that when drought is experienced during different developmental stages, selection might favour a tolerance strategy of photosynthetic alterations to survive drought during reproductive stages but might favour a strategy of rapid growth and reproduction to avoid stress at other times. This deduction is based on the observation that, in leaves, drought-specific gene expression dominates over stage-specific expression, whereas, stage-specific expression dominates over drought-specific expression in reproductive tissues. Furthermore, these selective genes with tissue-specific expression patterns were enriched in distinct pathways, suggesting that maize might have evolved to keep the drought-response pathway selectively inactive until needed so that it can conserve the energy that the drought response would require. If so, this ability to control when the pathway is “on” would provide important genetic resources for improvement of drought tolerance in maize, as well as in other crops.

## Materials and Methods

### Maize RNA-seq data sets

A total of 96 maize (*Zea mays*) RNA-seq data sets were generated from 24 experiments^23^, which were all repeated four times, producing samples from three tissues (leaf, ear and tassel) of maize B73 at four developmental stages (V12, V14, V18 and R1) under two environmental conditions (well-watered and drought). The raw reads of RNA-seq were downloaded from the NCBI’s GEO (Gene Expression Omnibus) database (GEO accession number: GSE71723). Detailed information about these 96 maize RNA-seq data sets can be found in the previous paper^23^. We note that two of the 96 RNA-seq data sets (one for well-watered R1 tassel and the other for drought-stressed R1 tassel) each containing relatively fewer genome-matched reads (<five million) were not included in our study. Raw reads were trimmed for removing low quality reads (mean quality score <20; reads length <20) by using Trimmomatic (version 0.36; http://www.usadellab.org/cms/?page=trimmomatic)^67^. All sequenced reads trimmed were aligned to the maize B73 reference genome sequences (RefGen_v3; AGPv3.29; ftp://ftp.ensemblgenomes.org/pub/release-29/plants/fasta/zea_mays/dna/) by using TopHat (version 2.1.1; https://ccb.jhu.edu/software/tophat/index.shtml)^29^. Unique read alignments in bam (Binary Alignment/MAP) format were inputted into Cufflinks (version 2.2.1; http://cole-trapnell-lab.github.io/cufflinks/)^30^ for normalization and estimation of gene expression level in terms of FPKM (fragments per kilobase per million). A gene was regarded as expressed in a sample if the FPKM value was greater than one under at least one experimental condition. The genome annotation of maize B73 used in these analyses was obtained from Ensembl Plants (ftp://ftp.ensemblgenomes.org/pub/release-29/plants/gtf/zea_mays).

### PCA and hierarchical clustering

Genes expressed in at least one sample were used to perform PCA with the *prcomp* function *(center* = *TRUE*, *scale* = *FALSE)* from the R package *stats* (https://stat.ethz.ch/R-manual/R-devel/library/stats/html/stats-package.html). The 3D diagram of PCA for all 24 samples was visualized with the R package *scatterplot3d* (version 0.3–37; https://cran.r-project.org/web/packages/scatterplot3d/index.html)^68^. The *summary* function in *prcomp* was used to calculate the proportion of variance in gene expression profiles explained by each principal component. For hierarchical clustering, a Pearson correlation was used to compute the similarities between gene expression profiles among different experiments using the R function *cor*, and the complete linkage method as well as the Euclidean distance measure were used for hierarchical clustering of gene expression profiles with the R function *hclust*. The correlation heatmap was plotted using the R function *corrplot*.

### Maize TFs annotation

The cDNA sequences of maize TFs and their corresponding family annotation information were downloaded from GRASSIUS (http://grassius.org/)^69^ and PlantTFDB (http://planttfdb.cbi.pku.edu.cn/)^56^. The cDNA sequences of maize TFs were then aligned to the maize B73 genome sequences using blat^70^. When a cDNA sequence was aligned in multiple places, the best alignment was screened using *pslSort* and *pslRep* functions in blat. Finally, maize TFs were annotated on the basis of the genomic coordinates and annotation information of cDNA sequences and the genome annotation of maize B73.

### Differential expression analysis

Pairwise differential expression analysis at the gene level was conducted using the Cuffdiff program in Cufflinks (version 2.2.1)^30^. Genes were regarded as differentially expressed (DE) using the cut-off criteria of a false discovery rate (FDR)-adjusted *P-value* ≤ 0.05 and a fold change ≥2 or ≤0.5. The distribution plots of DE genes were generated using the circular visualization tool Circos (version 0.69; http://www.circos.ca)^71^, in which lines represented the number of DE genes counted in a 1 Mbp window. DE genes detected from Cuffdiff were further verified by two R packages, DESeq^31^ and edgeR^32^. Aligned reads were counted using HTSeq. 0.7.2^72^, and the differential expression analyses with DESeq and edgeR were performed according to previously published protocols^73^.

### Gene co-expression network analysis

Gene co-expression network analysis was performed to group DE genes into modules using the R package WGCNA (version 1.49; https://labs.genetics.ucla.edu/horvath/CoexpressionNetwork/Rpackages/WGCNA/)^74^. In a gene co-expression network, a node corresponded to a DE gene; an edge was determined by the similarity between expression profiles of paired genes calculated with Pearson correlation. An adjacency matrix was built by applying a power function (*β*) on the Pearson correlation matrix. The *β* was optimized to be 24 for balancing the scale-free property of the co-expression network and the sparsity of connections between genes. The adjacency matrix was used to calculate the topographical overlap matrix (TOM), which measures the number of neighbours that a pair of genes have in common, relative to the rest of the genes. Hierarchical clustering was used to group genes on the basis of dissimilarity of gene connectivity, defined as 1–TOM. The *cutreeDynamic* function was used to produce co-expression clusters, with parameters as deepSplit = 2, pamRespectDendro = F, minClusterSize = 30. Highly correlated clusters were merged to form modules in the network using the *mergeCloseModules* function with cutHeight set to 0.25. For each module, hub genes were identified as the top 10% genes with the highest hub scores calculated by the igraph package^38^. Modules were visualized using Cytoscape (version 3.4.0; http://www.cytoscape.org/download.php)^75^, setting the layout with edge-weighted spring embedded layout. Circles were coloured on the basis of the corresponding module colours.

### AS identification

To identify AS events, firstly, the assembled transcript isoforms were merged together using the Cuffmerge module of the Cufflinks package, as described previously^76^. The transcripts of corresponding genes within each module were respectively used as the input for AStalavista (http://genome.crg.es/astalavista/)^77^ for identifying different AS events.

### Functional annotation of hub genes and modules

AgriGO (http://bioinfo.cau.edu.cn/agriGO)^78^ was used to perform GO term enrichment analysis of hub genes and modules. Using the maize whole genome as the background/reference, the statistical significance of GO terms was first evaluated with the Fisher’s exact test and then corrected using Yekutieli FDR adjustment. Only those GO terms with adjusted *P*-value below 0.05 and five or more query genes were determined to be statistically significant. The pathway maps were obtained from the KEGG database^79^ (http://www.genome.jp/kegg/pathway.html). The pathway enrichment analysis was performed by using KOBAS v2.0 (http://kobas.cbi.pku.edu.cn)^80^, and a Benjamini and Hochberg adjusted *P*-value of 0.05 was used as the cut-off criterion.

### Heatmap and tendency patterns of expression values

The log_2_-transformed FPKM values of genes in each module were clustered and visualized using the R function *pheatmap*. To avoid an infinite value, a value of 0.01 was added to the genes with FPKM value of zero before log_2_-transformation. The tendency patterns were constructed on the basis of scaled FPKM values for the expression of genes in the 24 experiments using the *scale* function.

### Syntenic blocks identification

The syntenic relationships of the maize genes were identified by comparing maize B73 genome sequences using the SynMap^51^ utility of the CoGe website (https://genomevolution.org/coge/). The syntenic blocks containing multiple genes were detected using APGv3 CDS data with default settings except for the Quota Align Merge algorithm, and the final syntenic gene-set output with GEvo links was downloaded for further analysis.

### Regression analysis

The regression analysis was performed using the R function *lm* on genes, the expression of which were changed by at least threefold among four developmental stages. The linear relationships between gene expression value and the vector c(0,1,2,3) were calculated. An R-squared value of more than 0.65 was defined as a significant expression change during development, as previously described^23^.

## Electronic supplementary material

Supplementary information

Table S1

Table S2

Table S3

Table S4

Table S5

Table S6

Table S7

Table S8

Table S9

Table S10

Table S11

Table S12

Table S13

Table S14

Table S15

Table S16
