# Supplementary material for: A systems approach to a spatio-temporal understanding of the drought stress response in maize
Source: Sci Rep. 2017 Jul 26;7:6590. doi: 10.1038/s41598-017-06929-y (PMC5529502; doi:10.1038/s41598-017-06929-y)
Supplement: Supplementary file 1 — Supplementary information [file 41598_2017_6929_MOESM1_ESM.pdf]

A systems approach to a spatio-temporal understanding of the drought stress response in maize

Zhenyan Miao[1, 2, 3†], Zhaoxue Han [1, 3†], Ting Zhang [1] , Siyuan Chen[1, 2] and Chuang Ma[1, 2, 3*]

[1]*State Key Laboratory of Crop Stress Biology for Arid Areas, College of Life Sciences, Northwest A&F University, Yangling 712100, Shaanxi, China.*

[2]*Center of Bioinformatics, College of Life Sciences, Northwest A&F University, Yangling 712100, Shaanxi, China.*

[3]*Key Laboratory of Biology and Genetics Improvement of Maize in Arid Area of Northwest Region, Ministry of Agriculture, Northwest A&F University, Shaanxi, Yangling 712100, China.*

***Correspondence:** Chuang Ma. E-mail: chuangma2006@gmail.com*

[†]*These authors contributed equally to this work.*

Supplementary information

Figure S1-S12

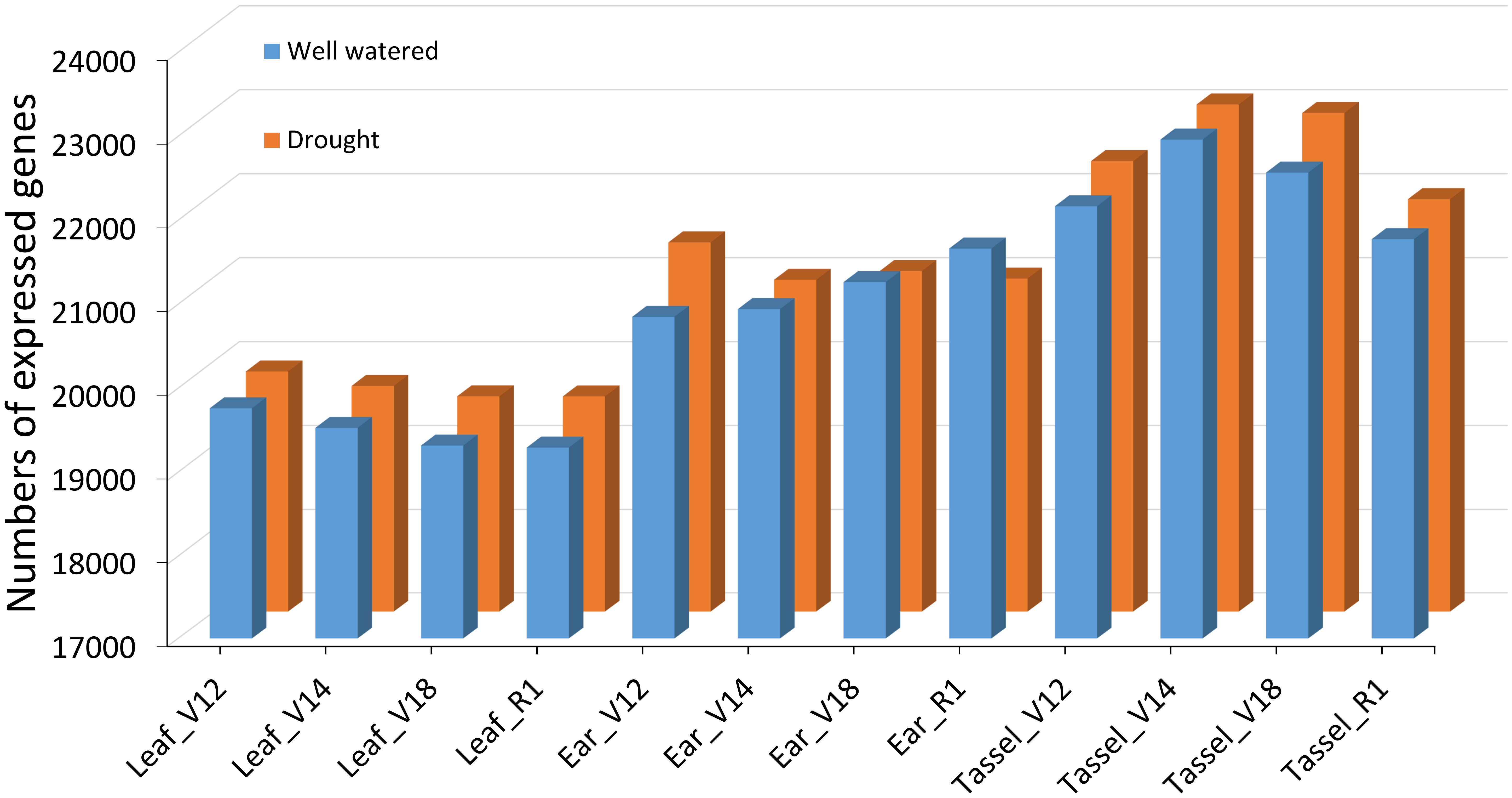

Figure S1 Number of expressed genes at four developmental stages (V12, V14, V18 and R1) in three tissues (leaf, ear and tassel) under two conditions (well-watered and drought).

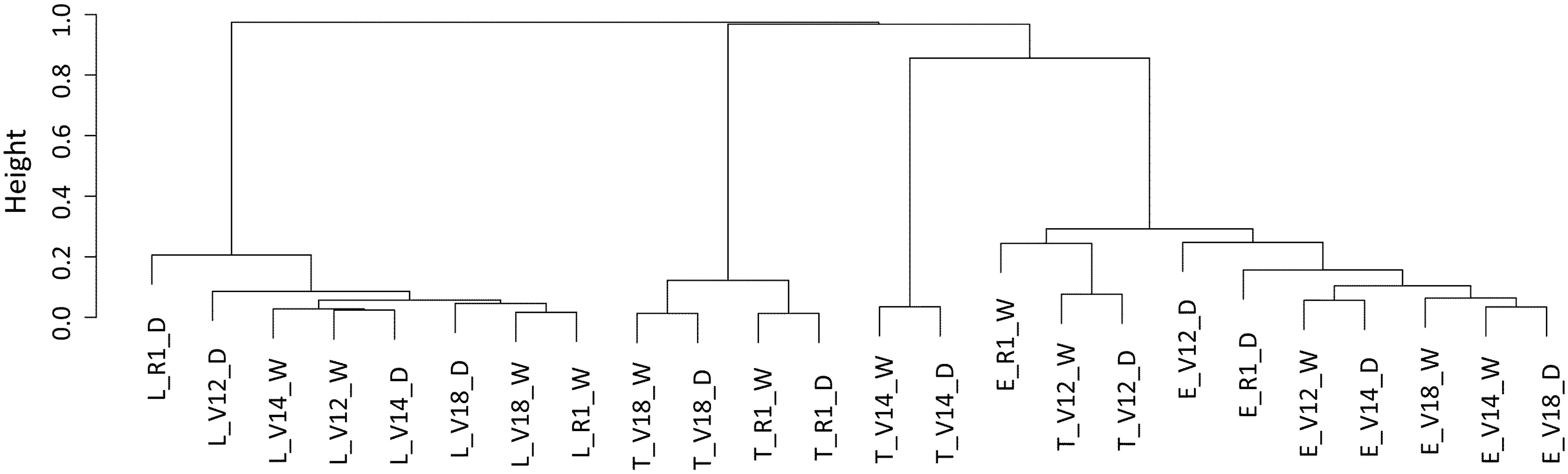

Figure S2 Hierarchical clustering of gene expression profiles of total 24 samples. L, leaf, T, tassel, E, ear, W, well-watered, D, drought.

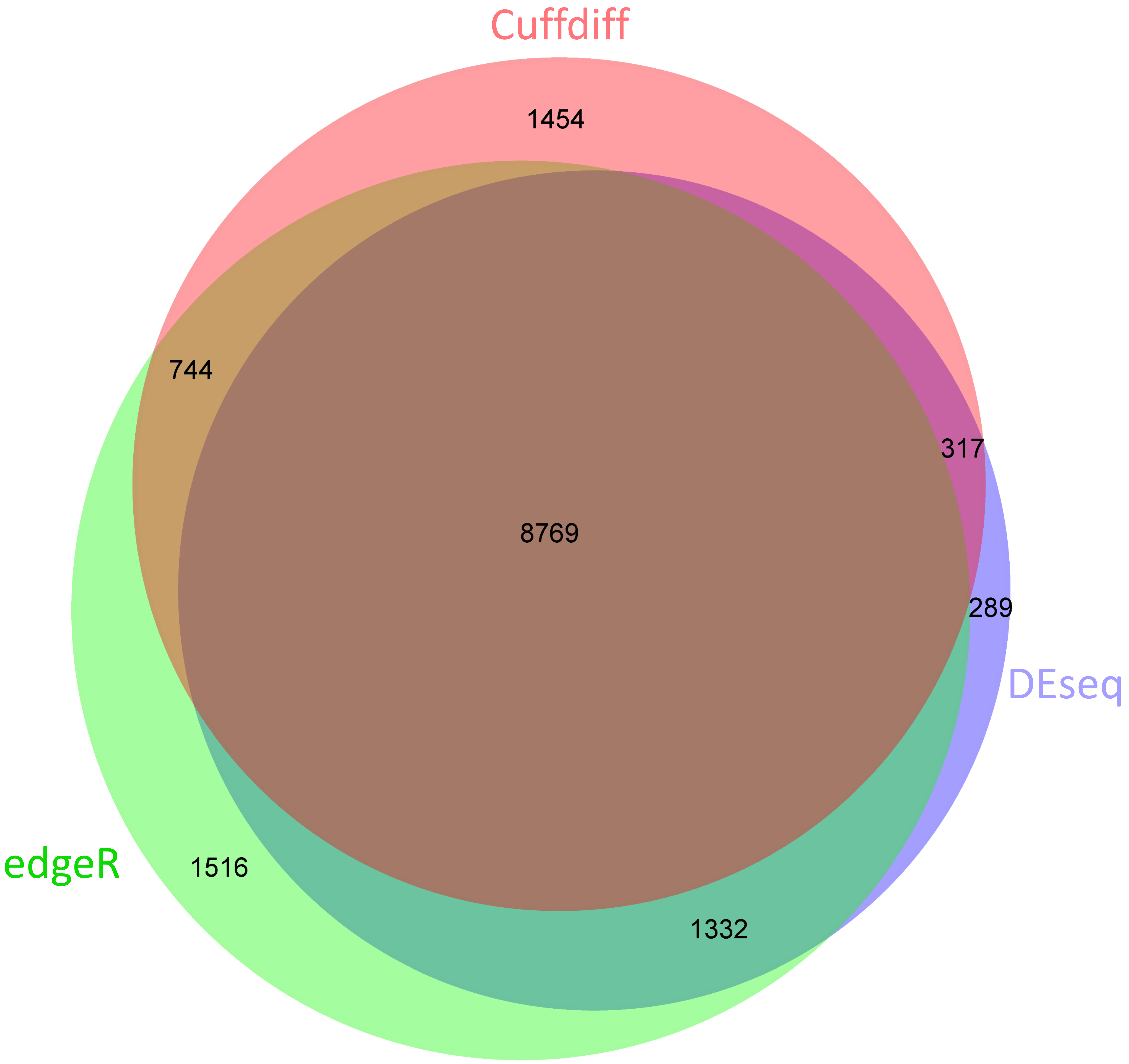

Figure S3 Shared and unique DE genes identified by Cuffdiff, DEseq and edgeR.

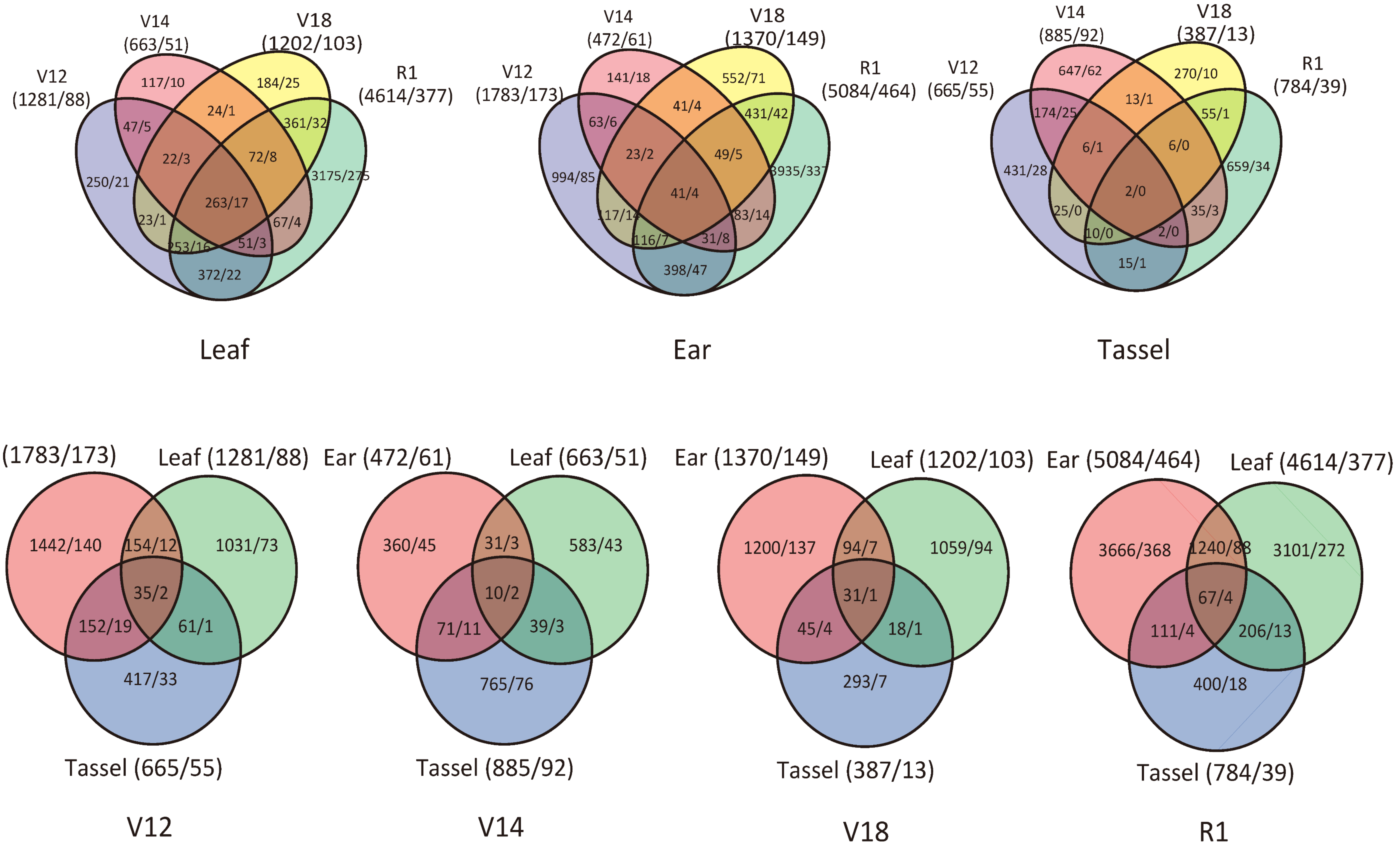

Figure S4 Shared and unique DE genes in three tissues and four developmental stages respectively. Numbers in parenthesis indicate genes/TFs.

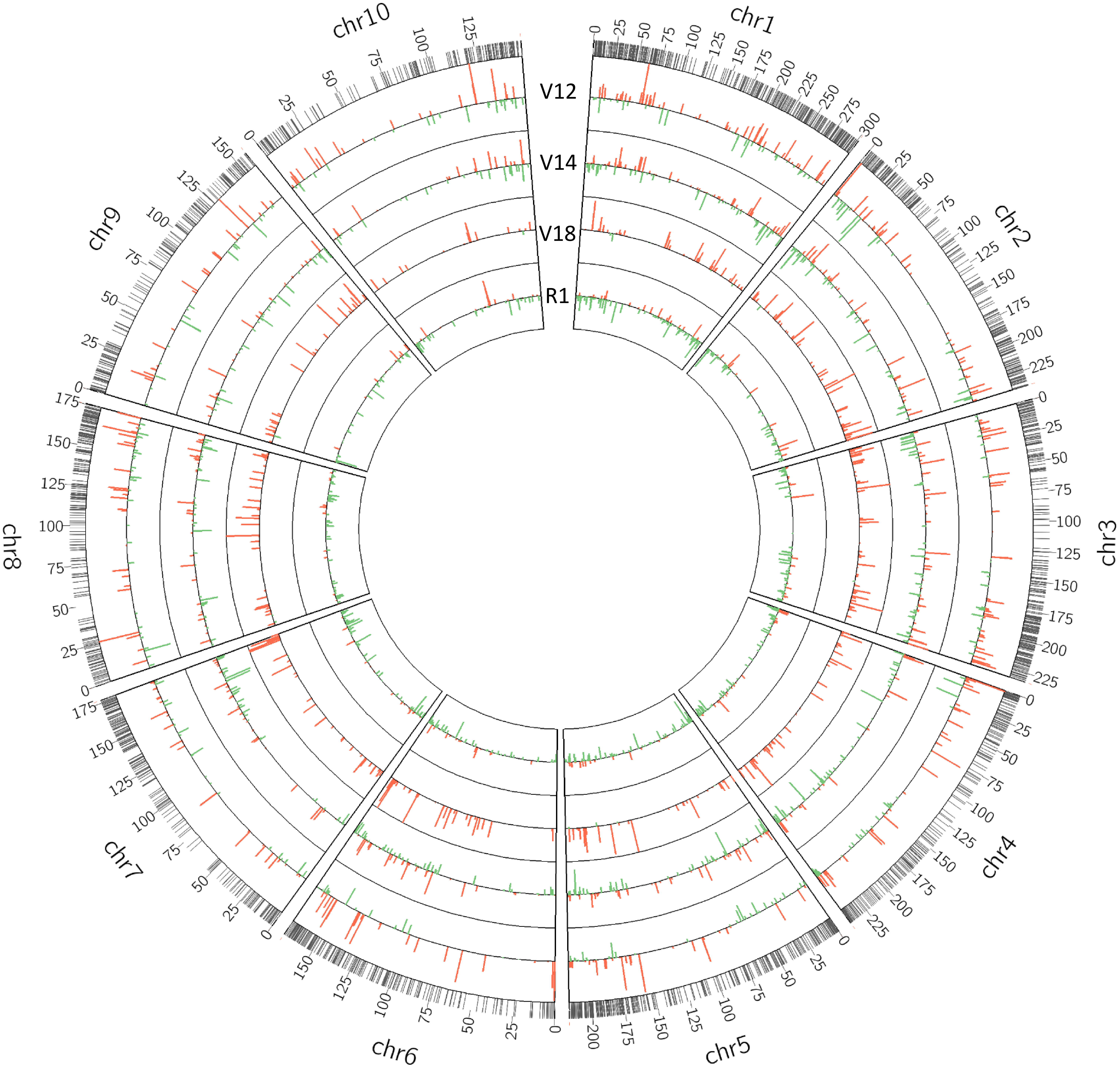

Figure S5 Distribution of DE genes at four developmental stages in tassel tissue. Outer track showed density of total DE genes in tassel. Red color indicates up-regulated by drought stress. Green color indicates down-regulated by drought stress.

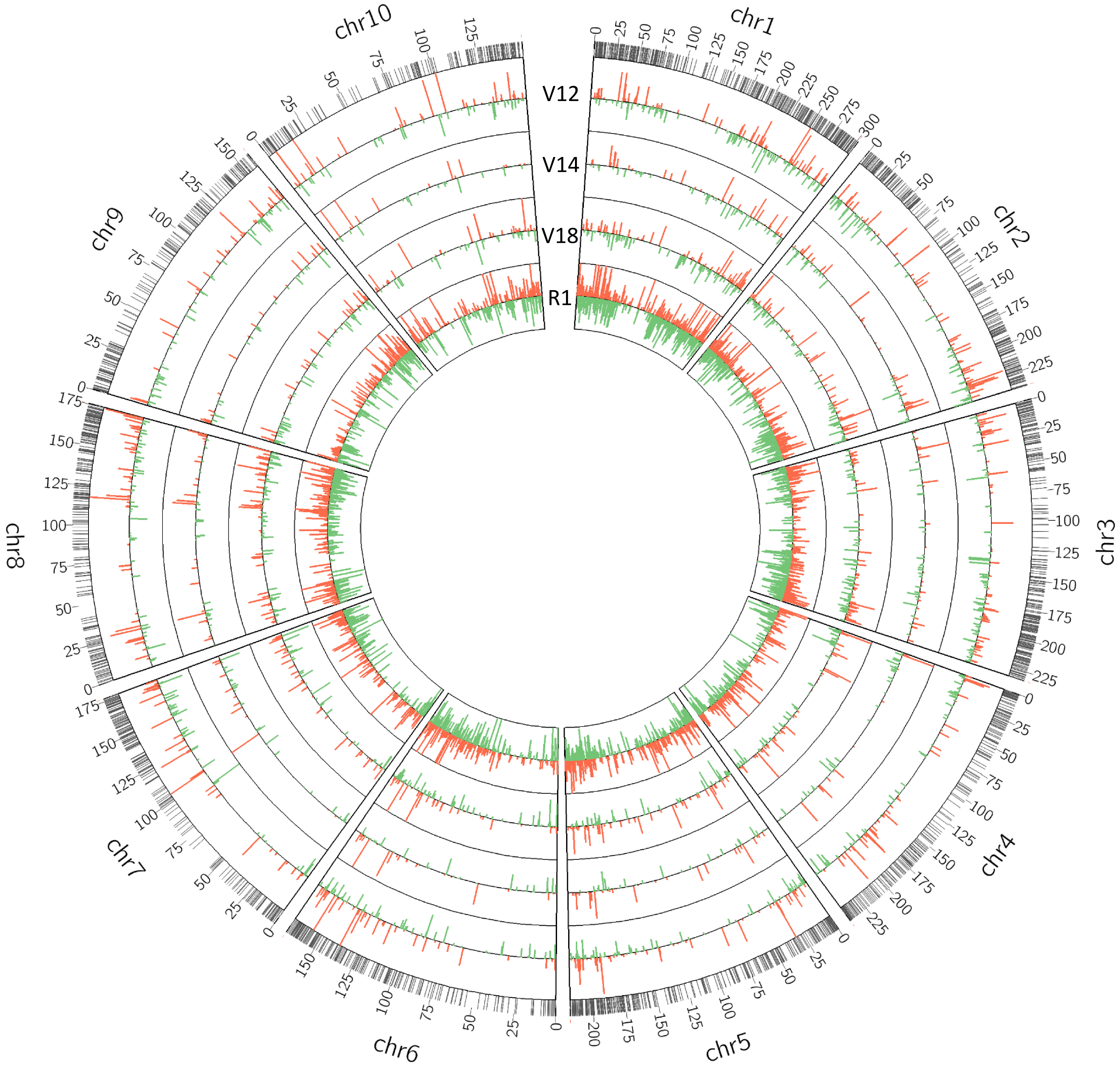

Figure S6 Distribution of DE genes at four developmental stages in leaf tissue. Outer track showed density of total DE genes in leaf. Red color indicates up-regulated by drought stress. Green color indicates down-regulated by drought stress.

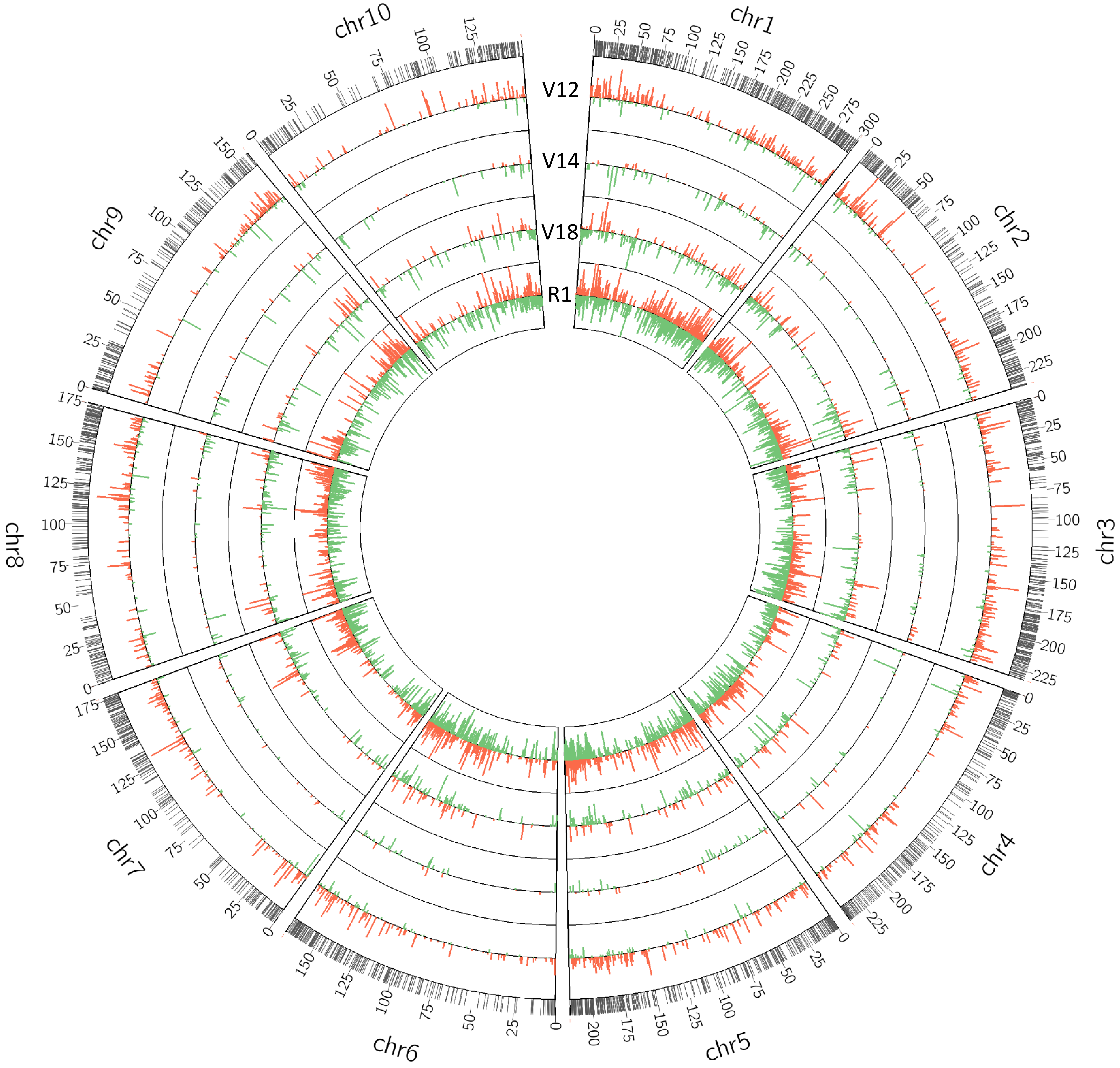

Figure S7 Distribution of DE genes at four developmental stages in ear tissue. Outer track showed density of total DE genes in ear. Red color indicates up-regulated by drought stress. Green color indicates down-regulated by drought stress.

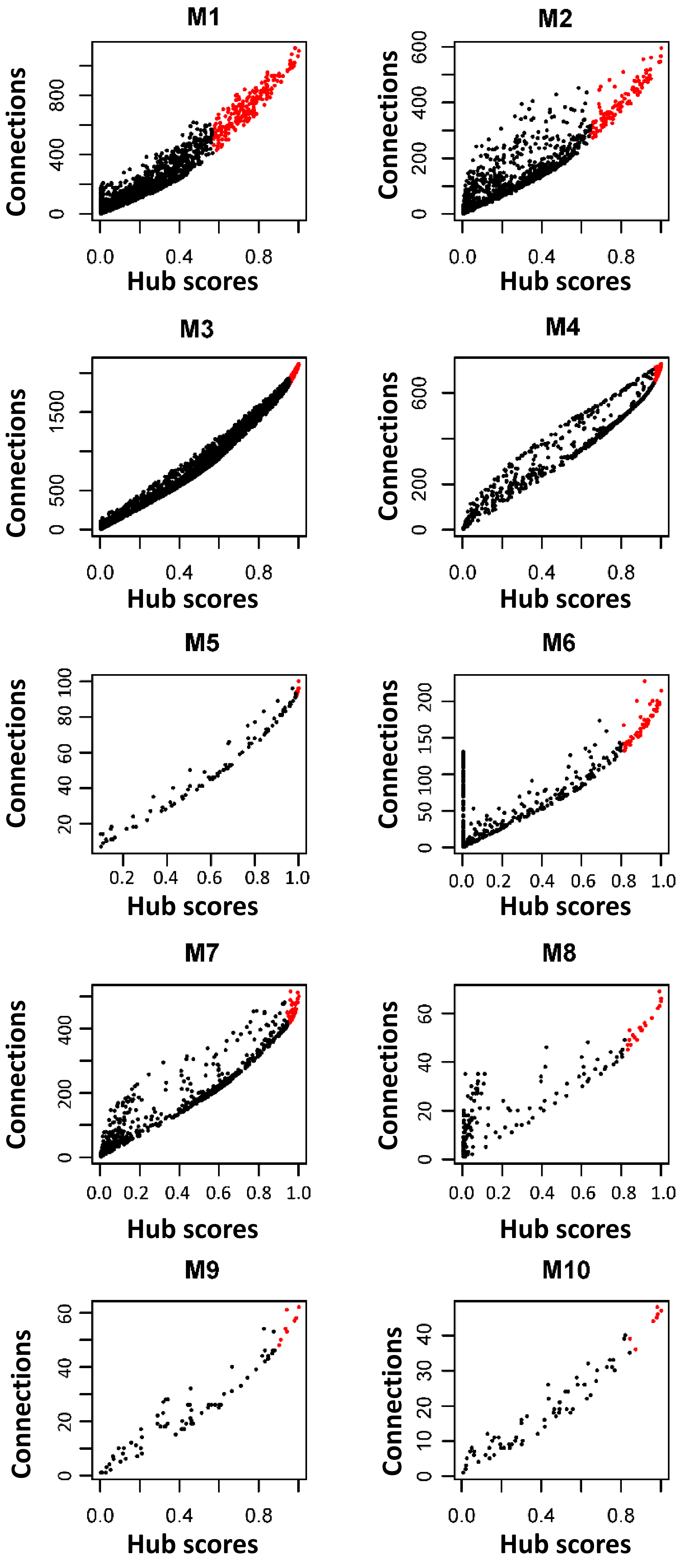

Figure S8 Scatter plots showed degree of connections (y axis) and hub scores calculated by igraph (x axis) of a given gene (node) in 10 modules. Red color indicated hub genes.

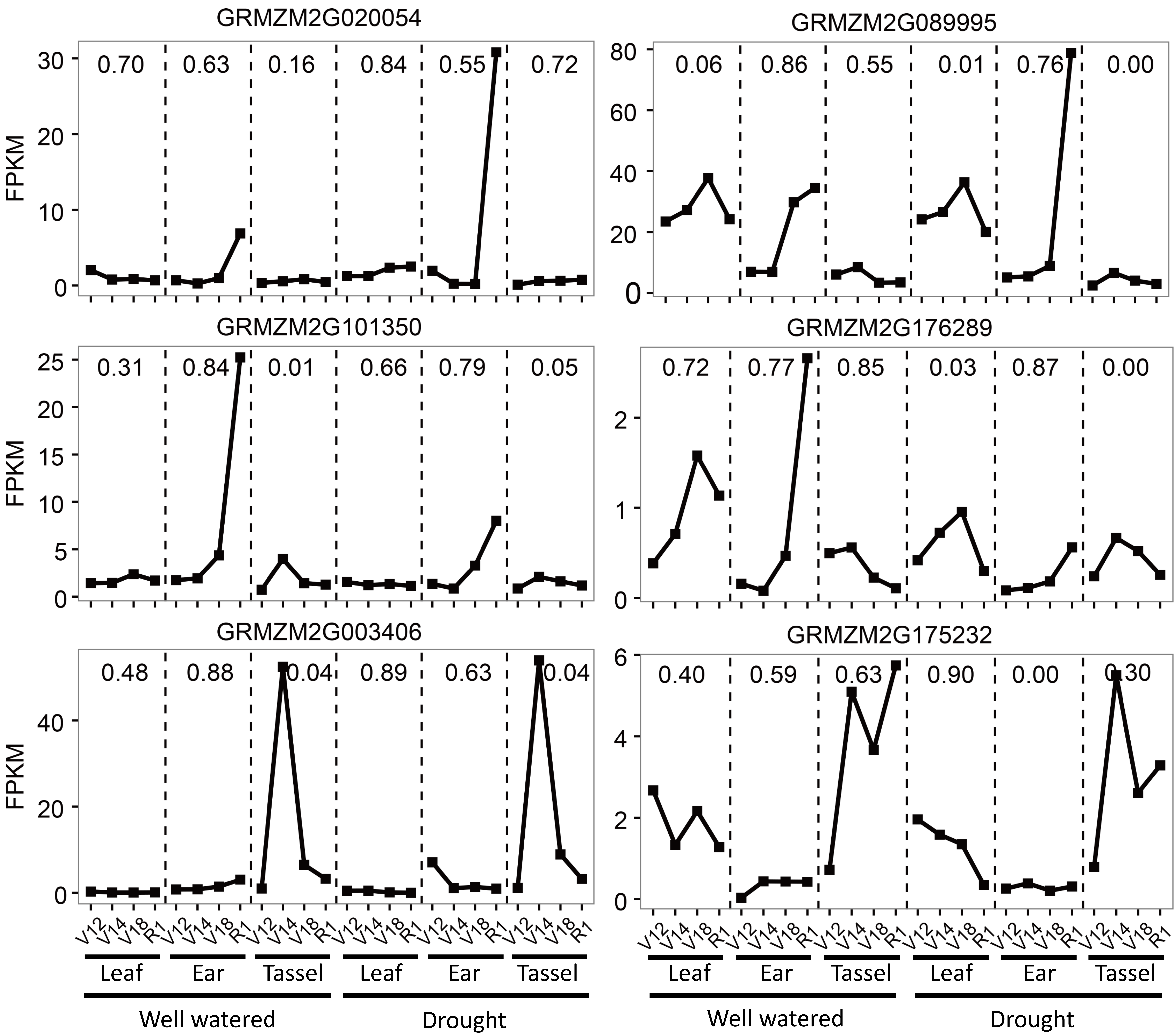

Figure S9 Significant expression changes of three TFs (GRMZM2G020054, GRMZM2G101350 and GRMZM2G003406) and corresponding duplicates over the course of development in leaf, ear and tassel under drought stress. Genes were considered significant if they changed by at least threefold and fit a linear or exponential regression curve at r2 > 0.65.

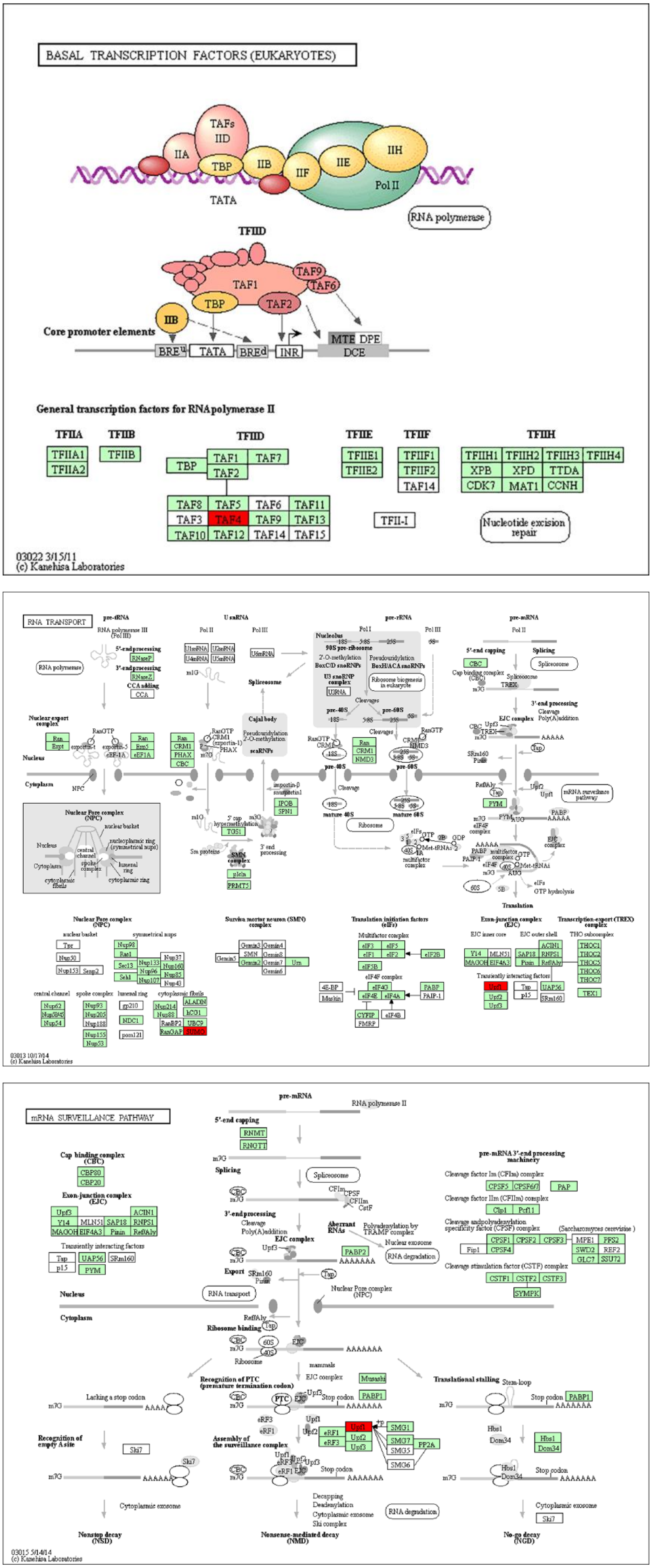

Figure S10 KEGG maps of three transcription related pathways (transcription initiation, RNA transport and mRNA surveillance). Red color indicated hub genes.

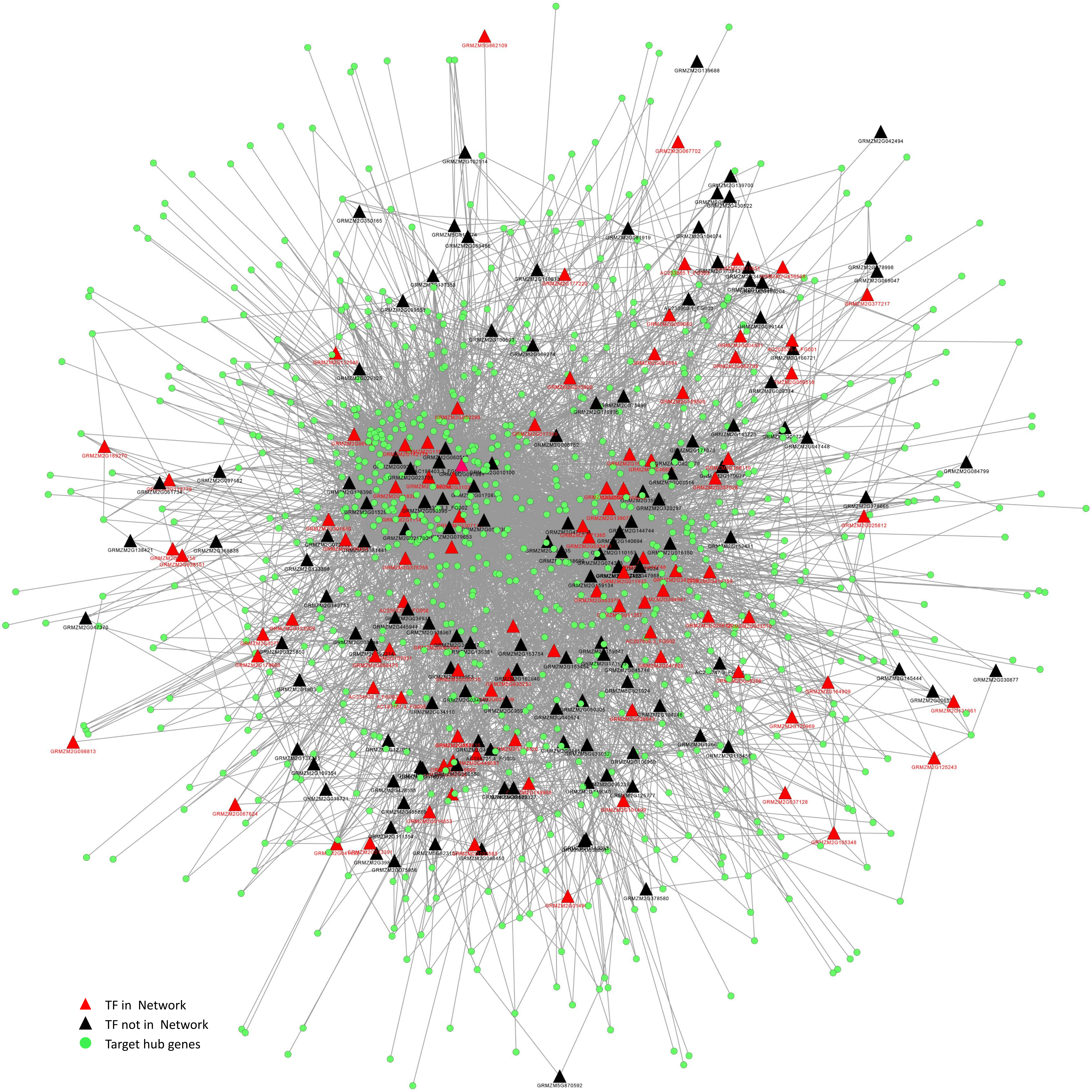

Figure S11 A topological overview of the regulatory network between each pair of TFs and targets. TFs are represented as triangles and targets are represented as ellipses. Detailed TF-target interactions are provided in Table S11.

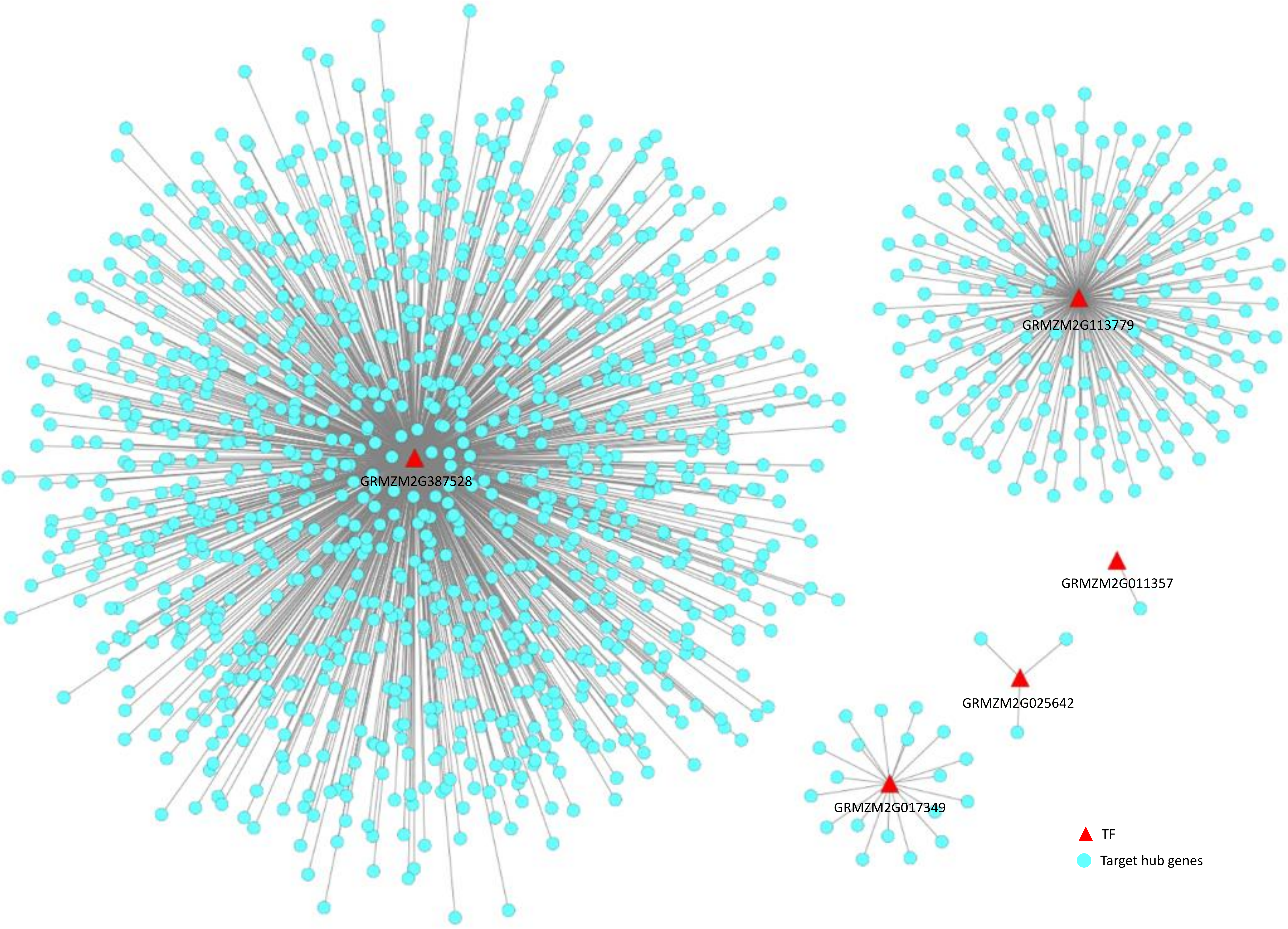

Figure S12 Five network TFs with over-presented targets. TFs are represented as triangles and targets are represented as ellipses.
